# Testing for COVID-19 is Much More Effective When Performed Immediately Prior to Social Mixing

**DOI:** 10.3389/ijph.2022.1604659

**Published:** 2022-07-27

**Authors:** Chad R. Wells, Senay Gokcebel, Abhishek Pandey, Alison P. Galvani, Jeffrey P. Townsend

**Affiliations:** ^1^ Center for Infectious Disease Modeling and Analysis (CIDMA), Yale School of Public Health, New Haven, CT, United States; ^2^ Yale School of Public Health, New Haven, CT, United States; ^3^ Grinnell College, Grinnell, IA, United States; ^4^ Department of Biostatistics, Yale School of Public Health, New Haven, CT, United States; ^5^ Program in Computational Biology and Bioinformatics, Yale University, New Haven, CT, United States; ^6^ Program in Microbiology, Yale University, New Haven, CT, United States

**Keywords:** RT-PCR, COVID-19, travel medicine, travel safety, rapid antigen test, post-arrival transmission, event safety

## Abstract

**Objective:** To quantify the utility of RT-PCR and rapid antigen tests in preventing post-arrival transmission based on timing of the pre-departure test.

**Methods:** We derived analytical expressions to compute post-arrival transmission when no test is performed, and when either an RT-PCR or any of 18 rapid antigen tests is performed at specified times before arrival. We determined the diagnostic sensitivity of the rapid antigen tests by propagating their RT-PCR percent positive agreement onto known RT-PCR diagnostic sensitivity.

**Results:** Depending on the rapid antigen test used, conducting a rapid antigen test immediately before departure reduces post-arrival transmission between 37.4% (95% CrI: 28.2%–40.7%) and 46.7% (95% CrI:40.0%–49.3%), compared to a 31.1% (95% CrI: 26.3%–33.5%) reduction using an RT-PCR 12 h before arrival. Performance of each rapid antigen test differed by diagnostic sensitivity over the course of disease. However, these differences were smaller than those engendered by testing too early.

**Conclusion:** Testing closer to arrival—ideally on the day of arrival—is more effective at reducing post-arrival transmission than testing earlier. Rapid antigen tests perform the best in this application due to their short turnaround time.

## Introduction

Travel has been greatly reduced during the COVID-19 pandemic, with declines in airfare following border closures and cancellation of flights due to safety concerns [1, 2]. Restrictions on gathering size have halted many festivals and events that provide human social activity and stimulate the economy. A widely adopted approach to limit transmission is to solely require a test for COVID-19 prior to arrival at the travel destination or at large events [3, 4].

Many countries have adopted such testing strategies to resume travel in a safer manner. For example, many European Union countries require a negative RT-PCR test 72 h prior to entry. In many cases, pre-departure testing may be coupled with testing and quarantine to ensure minimal imminent infection in the country of arrival [5]. Similarly, event organizers have adopted testing rules to prevent super-spreading events. For many events, attendees need either a proof of COVID-19 vaccination, or testing prior to event entry. However, there is no broad consensus for the timing of the test conducted prior to arrival. While testing 48–72 h prior is a common requirement for attendance or travel, longer time windows for testing are still allowed in some organizations. For example, events hosted by the US Track and Field Association require participants to undergo testing within the past 7 days [6]. Similarly, the National Archives and Records Administration requires employees who have failed to present their weekly COVID-19 test results to provide new test results, with the collection date no longer than a week prior to entry [7]. To assess the effectiveness of testing in identifying cases, many studies have evaluated the sensitivity of RT-PCR and antigen tests, and even aspects of the optimal time window for testing [8, 9]. Kucirka et al [8] report that testing for COVID-19 early in the incubation period is more likely to yield inaccurate results than when the test is conducted later on when symptoms appear. In the context of travel, projects developed prior to the pandemic—such as Prevention and Management of High Threat Pathogen Incidents in Transport Hubs (PANDHUB)—have used modeling approaches to assess mitigation approaches (e.g., screening to detect cases) for high-threat diseases (e.g., Ebola virus disease, pandemic influenza, and pneumonic plague) [10]. These methods developed for other diseases can be applied to inform disease control efforts for SARS CoV-2. Some of the travel related studies for SARS CoV-2 have qualitatively argued that the application of testing close to departure is desirable to detect and isolate infected individuals [11, 12]. Kiang et al. [12] contended that conducting a test within 3 days of departure, combined with a post-quarantine test following arrival, was most ideal in reducing onward transmission. Johansson et al. [11] use a mathematical model to show that the risk of transmission diminishes as the time between testing and departure shortens. Using Monte Carlo simulations, Clifford et al. [13] show that testing a day before travel provides a greater reduction in post-arrival transmission than testing four or 7 days prior to travel. Quilty et al. [14] use mathematical modeling to quantify the effectiveness of thermal screening—which would have constant sensitivity in detecting a case in comparison to the temporal sensitivity of diagnostic molecular tests—upon departure and arrival of airline passengers. However, no study uses a mathematical model to compare the effectiveness of pre-arrival testing in reducing transmission across multiple types of tests or has evaluated how pre-arrival testing impacts the probability of onward transmission after arrival. To aid in the safe resumption of travel and attendance at large gatherings, we determine the optimal timing for use of RT-PCR testing and 18 antigen tests prior to arrival. We derive analytical expressions for the expected post-arrival transmission of SARS-CoV-2 and compute the probability of onward transmission when 1) testing is not required prior to arrival and 2) testing conducted 1 week prior to arrival up to the time of arrival. We focus our analysis on the RT-PCR test and five popular rapid antigen tests (BD Veritor, BinaxNOW, CareStart, LumiraDx and Sofia), while also examining an additional 10 brands of antigen tests that have received Emergency Use Approval from the US Food and Drug Administration.

## Methods

### Transmission Over Time

Transmission of a pathogen from an infected individual is typically time-dependent, based on pathogen shedding and behavioral changes, and can be represented over time by a function r(t) , for which time *t* = 0 represents initial infection. To represent infectiousness of an individual, a function r(t) can be scaled such that

$$\int_{t\;=\;0}^{\infty }r\left(t\right)dt=R_{0}\text{,}$$ (1)

where $R_{0}$ is the basic reproduction number: the expected number of infections consequent to a single infected individual under a scenario of no intervention. Specifying a discrete end to the infection at time $t_{e}$ (i.e., 20 days after symptom onset [15–17]) such that r(t)=0 for $t>t_{e}$ ,

$$\int_{t\;=\;0}^{t_{e}}r\left(t\right)dt=R_{0}\text{.}$$

### Self-Isolation at Symptom Onset

A significant means of intervention to prevent infection is self-isolation of infected individuals upon symptom onset. We express the transmission over time for a symptomatic individual who isolates upon symptom onset as,

$$r_{S}\left(t\right)=\{\begin{array}{cc} r\left(t\right) & \mathrm{if}\;0\leq t\leq t_{S},\\ 0 & \mathrm{if}\;t>t_{S} \end{array}.$$

Specifying a proportion $p_{a}$ of infected individuals who can infect others but never manifest symptoms (i.e., that are asymptomatic carriers), then transmission may be partitioned into the contributions of symptomatic and asymptomatic cases as $R_{0}=R_{0,s}p_{s}+R_{0,a}p_{a}$ , in which the probability of a symptomatic case $p_{s}=\left(1-p_{a}\right)$ . $R_{0,s}$ and $R_{0,a}$ can be equated to distinct infectiousness functions $r_{s}\left(t\right)$ and $r_{a}\left(t\right)$ , in the absence of self-isolation. Concordant with previous research [18, 19], we set $R_{0,s}=\;R_{0,a}$ and equivalent infectivity profiles in the absence of self-isolation (i.e., $r_{s}\left(t\right)=r_{a}\left(t\right)=r\left(t\right)$ ). If asymptomatic cases are less infectious than symptomatic cases, this assumption of equivalency entails that our estimate of the post-arrival transmission is an upper bound. Alternate overall transmission and alternate forms of infectivity over time for asymptomatic cases may easily be partitioned and tracked in the theory that follows should there be evidence to substantiate their difference.

The presence of asymptomatic carriers increases the degree of transmission consequent to a self-isolation intervention such that

$$R=p_{s}\int_{t\;=\;0}^{t_{s}}r_{S}\left(t\right)dt+p_{a}R_{0}\;$$ (2)

### Pre-Departure Testing

In a rapidly spreading epidemic, individuals who might be traveling or attending events will tend to be early in disease time-course. In a rapidly declining epidemic, individuals who might be travelling or attending events will tend to be later in disease time-course. In a steady-state epidemic with case counts c(t) , the change in case counts $\frac{dc}{dt}\;\approx \;0$ over the period from the time of infection to *t*_s_, such that asymptomatic or presymptomatic individuals who might be about to travel or about to participate in an event are uniformly distributed across the disease time course. Provided all individuals experience symptoms at time $t_{s}$ and that those experiencing symptoms are excluded from arrival at time *t*_*s*_, then the expected post-travel transmission from an infected individual is

$$R_{v^{\to }}\left(v\right)=\int_{t=v}^{\infty }r_{S}\left(t\right)dt,$$

where *v* is the time of arrival to an event or travel destination relative to the start of infection.

To evaluate the impact of a pre-arrival test on onward transmission from infected individuals based on when the test is administered, we can account separately for those individuals who are infected subsequent to the test and prior to travel, and those individuals who are infected prior to the test and prior to travel.

If testing occurs *w* days prior to travel, individuals who are infected subsequent to the test and prior to arrival who will exhibit symptoms and self-isolate at time *t*_*s*_—will contribute

$$\int_{t=w-u}^{t_{s}}r_{S}\left(t\right)dt,$$

where *u* is the duration between the test and time of infection. Integrating uniformly over potential times of infection of an individual subsequent to the test and prior to arrival, the expected contribution to post-arrival transmission for a symptomatic case is

$$R_{S,w^{\to }}\left(w\right)=\frac{1}{w\;}\int_{u=0}^{w}\int_{t=w-u}^{t_{s}}r_{S}\left(t\right)dt\;du\text{.}$$ (3)

In contrast, individuals who are infected prior to the test (and prior to arrival)—and who will exhibit symptoms and self-isolate at time *t*_*s*_—will contribute

$$\int_{t=u+w}^{t_{s}}\left(1-s\left(u\right)\right)\cdot \;r_{S}\left(t\right)\;dt\text{,}$$

where *u* is the duration from time of infection to the time the test is conducted, and s(u) is the time-dependent diagnostic sensitivity of the test. Integrating uniformly over potential times of infection of an individual infected prior to the test, the expected contribution to post-arrival transmission for a symptomatic case is

$$R_{S,x^{\mapsto }}\left(w\right)=\frac{1}{t_{s}-w\;}\int_{u=0}^{t_{s}-w}\int_{t=u+w}^{t_{s}}\left(1-s\left(u\right)\right)\cdot \;r_{S}\left(t\right)dt\;du\text{.}$$ (4)

Because Eqs 3, 4 quantify expected contributions to post-arrival transmission consequent to mutually exclusive and exhaustive events, the total post-arrival transmission from an infected traveler who will manifest symptoms as a function of when the pre-arrival test is administered is

$$R_{S,T^{\mapsto }}\left(w\right)=\frac{w}{t_{s}}R_{S,w^{\mapsto }}\left(w\right)+\frac{t_{s}-w}{t_{s}}R_{S,x^{\mapsto }}\left(w\right)\text{.}$$ (5)

Individuals who are infected subsequent to the test and prior to arrival—and who will not exhibit symptoms or self-isolate without a positive test result—will contribute

$$R_{A,w^{\mapsto }}\left(w\right)=\frac{1}{w\;}\int_{u=0}^{w}\int_{t=w-u}^{t_{e}}r_{A}\left(t\right)dt\;du\text{,}$$ (6)

where *t*_*e*_ is the time from infection until cessation of infectivity. In contrast, individuals who are infected prior to the test (and prior to arrival at event or travel destination)—and who will not exhibit symptoms or self-isolate without a positive test—will contribute

$$R_{A,x^{\mapsto }}\left(w\right)=\frac{1}{t_{e}-w\;}\int_{u=0}^{t_{e}-w}\int_{t=u+w}^{t_{e}}\left(1-s\left(u\right)\right)\cdot \;r_{A}\left(t\right)dt\;du\text{,}$$ (7)

in which the time-dependent diagnostic sensitivity of the test, in time *t* since infection, is s(t) .

Because Eqs 6, 7 quantify expected contributions to post-arrival transmission consequent to mutually exclusive and exhaustive events, the total post-arrival transmission from infected travelers who will not manifest symptoms as a function of when the pre-arrival test is administered is

$$R_{A,T^{\mapsto }}\left(w\right)=\frac{w}{t_{e}}R_{A,w^{\mapsto }}\left(w\right)+\frac{t_{e}-w}{t_{e}}R_{A,x^{\mapsto }}\left(w\right)\text{.}$$ (8)

Incorporating both symptomatic and asymptomatic infections, **Eqs 5**, **8** are exhaustive and exclusive at respective proportions of non-isolated infection *p*_*S*_ and *p*_*A*_, the total post-arrival transmission from infected travelers as a function of when the pre-arrival test is administered *w* days before arrival is

$$R_{T^{\mapsto }}\left(w\right)=p_{S}R_{S,T^{\mapsto }}\left(w\right)+p_{A}R_{A,T^{\mapsto }}\left(w\right)\text{.}$$ (9)

### Model Parameterization

Analytical expressions for the expected post-arrival transmission are informed by diagnostic performance data for RT-PCR and antigen testing, the timing of the test, the incubation period, the transmission over the disease time course [20], the basic reproduction number, and the proportion of asymptomatic infections [21].

Our computations use a RT-PCR diagnostic sensitivity curve that was constructed by piecewise mapping using the Cartesian pairing of the relative infectivity—obtained by dividing the infectivity profile by the magnitude of the peak infectivity—to diagnostic sensitivity from the pre- and post-peak infectivity (Supplementary Figure S1). Specifying an incubation period and corresponding distribution from Ashcroft et al [22], the baseline RT-PCR diagnostic sensitivity curve was obtained from a log-Normal distribution functional form [23] fit to the serial testing data of 27 healthcare workers from Hellewell et al [24] using a maximum likelihood approach [23, 24]—restricting the time of peak diagnostic sensitivity to the time of peak infectivity (Supplementary Figure S1). The functional form of this RT-PCR diagnostic sensitivity curve and distribution of the incubation period differs from that used in Hellewell et al [24]. From our RT-PCR diagnostic sensitivity curve, we constructed diagnostic sensitivity curves for each rapid antigen test using their temporal percent positivity agreement with RT-PCR—indicating that the diagnostic sensitivity of the rapid antigen can be no higher than that of RT-PCR. Specifically, the diagnostic sensitivity of the rapid antigen test at time *t* is determined by multiplying the diagnostic sensitivity of the RT-PCR test at time *t* by the percent positive agreement at time *t*. For each rapid antigen test, a linear logit model was fitted to the percent positive agreement data with a RT-PCR test from the time of symptom onset using a maximum likelihood approach [23]. To determine the percent positive agreement of the rapid antigen test during the incubation period, we used an interpolation function of the infectivity based on the Cartesian pairing of the infectivity and the percent positive agreement.

We specified an incubation period of 3.1 days [25] and basic reproduction number *R*_0_ = 6.93 [26,27] appropriate for the Omicron variant of concern (B.1.1.529), for our baseline analysis. To examine the impact of the incubation period on our results, we calculated the probability of post-arrival transmission for a RT-PCR diagnostic sensitivity curve that was informed by an incubation period of 4.4 days [28] and *R*_0_ = 5.08 [27]—appropriate for the Delta variant of concern (B.1.617.2)—as well as an incubation period of 5.72 days [22] and *R*_0_ = 2.79 [27]—appropriate for the original SARS-CoV-2 strain.

To account for over-dispersion of COVID-19 transmission [29], we specified the expected post-arrival secondary cases *R* to be negative-binomially distributed as

$$f\left(x|k,p\right)=\frac{\Gamma \left(k+x\right)}{\Gamma \left(k\right)\Gamma \left(x+1\right)}p^{k}{\left(1-p\right)}^{x}\text{,}$$

with the negative-binomial dispersion parameter *k* = 0.25 ^30,31^ and the negative binomial parameter *p* = *k*/(*k* + *R*); the negative binomial distribution is commonly used to model the overdispersion of secondary infections that is typically seen in the transmission of infectious disease [32]. Accordingly, the probability of post-arrival transmission was calculated as 1−f(0|k,p). To examine the impact of the dispersion parameter on our results, we conducted a one-way sensitivity analysis for a broad range of dispersion between 0.04 and 1 [29, 33].

To construct the credible intervals (2.5th and 97.5th percentiles), we first conducted a grid search, computing a broad and densely-populated likelihood surface for the parameterization of each diagnostic test. We then used likelihood-based importance sampling to obtain 1,000 importance-sampled parameter sets from values evaluated in the grid search. The proportion of infections that would remain asymptomatic across the time course of disease was obtained by drawing 1,000 samples from a Beta distribution—with a mean of 35.1%—calibrated to the 95% credible interval from 30.7% to 39.9% [21]. Computations were done in MATLAB, with source files available in an online repository [34].

## Results

Specifying an incubation period of 3.1 days [25] and 35.1% of infections remaining asymptomatic over the entire course of disease [21], we computed the reduction in the expected post-arrival transmission when testing is performed using either a RT-PCR test or one of the 18 commercially available rapid antigen tests up to 7 days prior to arrival relative to the expected post-arrival transmission when there is no testing**.**

### No Testing

**FIGURE 1:**
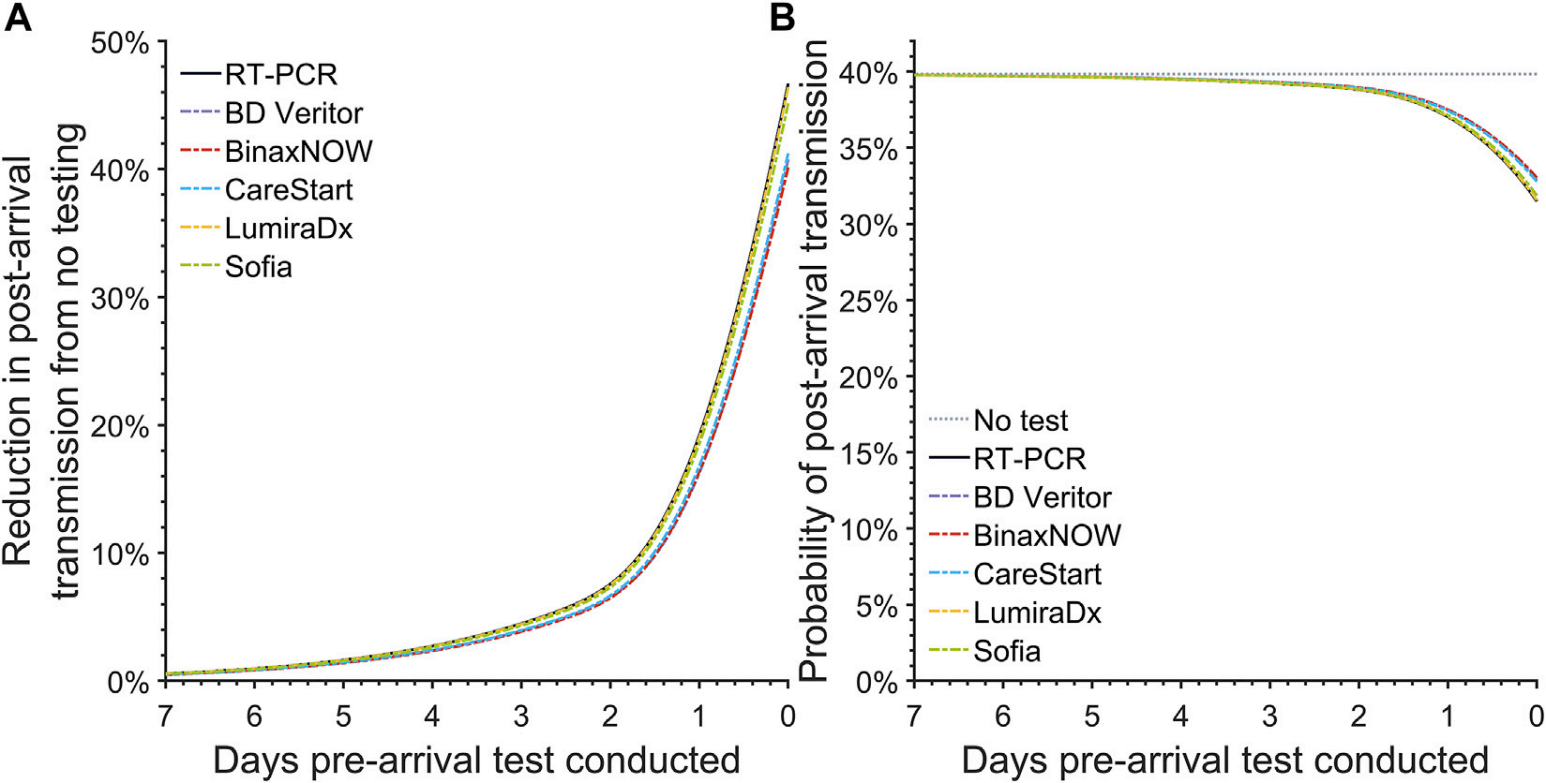
The post-arrival transmission for pre-arrival testing. Specifying an incubation period of 3.1 days, an RT-PCR diagnostic sensitivity based on data from Hellewell et al [24], and 35.1% infections as asymptomatic throughout disease, we calculated **(A)** the reduction in the post-arrival transmission compared to no pre-departure testing and **(B)** the probability of post-arrival transmission for a basic reproduction number of 6.93 when there is no pre-departure testing (gray dotted line), or when there is pre-departure testing using an RT-PCR test (black solid line), the BD Veritor (purple dashed line), BinaxNOW (red dashed line), CareStart (blue dashed line), LumiraDx (yellow dashed line), and Sofia (green dashed line) rapid antigen test conducted at departure or any previous day, up to 7 days prior to departure (United States, 2021–2022).

For a baseline reference, we computed the expected post-arrival transmission in the absence of testing. In the absence of pre-arrival testing, the probability of post-arrival/onward transmission is 39.8% (95% CrI: 39.6%–40.1%) (Figure 1B).

### Pre-Departure RT-PCR Testing

The reduction in expected post-arrival transmission became greater as the RT-PCR test was conducted closer to the day of arrival (Figure 1A). Accounting for a 24-h delay in obtaining results for RT-PCR tests, the expected post-arrival transmission for the RT-PCR test taken 24 h before departure declines by 19.2% (95% CrI: 16.0%–21.1%) (Figure 1A; Table 1). Reducing this turnaround time of 24 h to 12 h and testing 12 h before travel can decrease the expected transmission (Figure 1A), resulting in a decrease of the probability of post-arrival transmission from 37.0% (95% CrI: 36.5%–37.7%) to 34.9% (95% CrI: 34.3%–35.9%). If delays to obtain RT-PCR test results take as long as 72 h, then the probability of onward transmission can be as high as 39.2% (95% CrI: 38.9%–39.6%), with only a 4.5% (95% CrI: 3.6%–5.1%) reduction in the expected post-arrival transmission (Figure 1; Table 1). Testing a week prior to arrival at an event or travel destination produces a trivial 0.57% (95% CrI: 0.45%–0.65%) reduction in expected post-travel transmission (Figure 1A; Supplementary Figure S4).

**TABLE 1:** Reduction in expected post-arrival transmission after pre-arrival testing relative to no testing *^a^* (United States, 2021–2022).

Hours pre-departure	RT-PCR	BD Veritor	BinaxNOW	CareStart^b^	LumiraDx^b^	Sofia
72	4.5% (3.6%–5.1%) ^c^	3.9% (3.0%–4.6%)	3.9% (3.0%–4.5%)	4.0% (3.1%–4.7%)	4.5% (3.5%–5.1%)	4.3% (3.4%–5.0%)
48	7.6% (6.1%–8.6%)	6.5% (5.0%–7.7%)	6.5% (5.1%–7.6%)	6.7% (5.2%–7.9%)	7.5% (5.9%–8.6%)	7.3% (5.8%–8.5%)
24	19.2% (16.0%–21.1%)	16.2% (12.8%–18.6%)	16.4% (13.3%–18.5%)	16.8% (13.3%–19.3%)	19.0% (15.7%–21.1%)	18.5% (15.2%–20.8%)
12	31.1% (26.3%–33.5%)	26.7% (21.2%–29.9%)	26.6% (22.1%–29.7%)	27.4% (22.3%–30.8%)	30.9% (25.9%–33.5%)	30.1% (25.0%–33.0%)
0	46.7% (40.1%–49.3%)	40.8% (32.2%–44.8%)	40.1% (33.5%–44.0%)	41.2% (34.3%–45.6%)	46.4% (39.4%–49.2%)	45.1% (37.8%–48.5%)

a These computations are based on the RT-PCR diagnostic sensitivity curve constructed using data from Hellewell et al. [24] with the 95% credible intervals based on 1,000 samples of parameters determining diagnostic sensitivity and the proportion of infections that are asymptomatic.

b Anterior nasal swab.

c 95% credible interval.

### Pre-Departure Rapid Antigen Testing

We conducted analyses of 18 commercially available rapid antigen tests, focusing on the frequently used tests BD Veritor, BinaxNOW, CareStart, LumiraDx, and Sofia. Similar to RT-PCR, pre-arrival testing with rapid antigen tests led to a lower expected post-arrival transmission when testing was conducted closer to the time of arrival (Figure 1). Among the five most popular antigen tests, the reduction in the expected post-arrival transmission when testing at arrival ranged from 40.1% (95% CrI: 31.4%–42.5%) to 46.4% (95% CrI: 39.4%–49.2%), with LumiraDx exhibiting the best performance. When testing at arrival, the associated probability of post-arrival transmission for these five tests ranged from 31.5% (95% CrI: 30.7%–33.2%) to 33.0% (95% CrI: 32.5%–34.9%) (Figure 1B). Among all available antigen tests, the median reduction in the expected post-arrival transmission at departure was 45.2% (95% CrI: 38.7%–47.6%) with a range of 37.4% (95% CrI: 28.2%–40.7%) to 46.7% (95% CrI:40.0%–49.3%) (Supplementary Table S1); the median probability of post-arrival transmission was 31.8% (95% CrI: 31.1%–33.4%) (Supplementary Table S2). Of the 18 FDA-approved rapid antigen tests that could be conducted at time of arrival, none were found to result in greater transmission than RT-PCR conducted 24 h or 12 h before arrival (Supplementary Table S2).

### Scenario Analysis

We examined the impact of the incubation period on the effectiveness of pre-arrival testing in the reduction of post-arrival transmission relative to no testing by lengthening the incubation from 3.1 days [25] to 4.4 days [28] and 5.72 days [22]. The expected post-arrival transmission in the context of longer incubation periods is greater than that for shorter incubation periods. For example, specifying no test prior to arrival, the post-arrival transmission is 20.0% (95% CrI: 19.7%–20.3%) larger for the 4.4-day incubation relative to the post-arrival transmission under the 3.1-day incubation period. Specifying the 5.72-day incubation period, post-arrival transmission with no test is 35.9% (95% CrI: 35.4%–36.3%) greater than that for the 3.1-day incubation period. We found that the effectiveness of testing at arrival decreased very modestly for the longer incubation periods (Supplementary Figures S2, S3; Supplementary Tables S1, S3, S5). For example, conducting the BD-Veritor test at arrival, the reduction in post-arrival transmission declined from 40.8% (95% CrI: 32.2%–44.8%) to 39.9% (95%CrI: 31.5%–43.7%) and 39.3% (95% CrI: 31.0%–43.2%), respectively for the 4.4-day and 5.72-day incubation period. In contrast, the effectiveness of testing prior to arrival increased moderately when the incubation period was lengthened (Supplementary Figures S2, S3; Supplementary Tables S1, S3, S5). For example, an RT-PCR test conducted 12 h prior to arrival reduced post-arrival transmission by 31.1% (95% CrI: 26.3%–33.5%), increasing to 34.0% (95% CrI: 28.8%–36.4%) and 35.7% (95% CrI: 30.4%–38.2%) respectively for the 4.4-day and 5.72-day incubation periods. Under these different scenarios, a rapid antigen test performed at arrival still outperformed an RT-PCR that was conducted 12 or more hours prior to arrival (Supplementary Figures S2, S3; Supplementary Tables S1, S3, S5).

To compare the effectiveness of testing on arrival across different variants, we computed the probability of post-arrival transmission for Omicron, Delta, and the original variant. Using variant-specific incubation periods and basic reproduction numbers, we found that the probability of post-arrival transmission was greatest for the Omicron variant, and lowest for the original variant (Supplementary Figures S2, S3; Supplementary Tables S2, S4, S6). For all three variants, conducting RT-PCR 12 h prior to arrival was outperformed by all 18 rapid antigen tests when conducted at arrival (Supplementary Tables S1–S6).

To examine the impact of the negative-binomial dispersion parameter on the probability of post-arrival transmission, we conducted a one-way sensitivity analysis across a wide range from 0.04 to 1 for no testing, RT-PCR testing 12-h prior to arrival, as well as BD Veritor, BinaxNOW, CareStart, LumiraDx, and Sofia conducted on arrival [29, 33]. The probability of post-quarantine transmission increases with the dispersion parameter, with the differences between tests becoming magnified (Supplementary Figure S5).

## Discussion

Here we have quantified the effect of COVID-19 testing before large-scale events or travel on curtailing subsequent infections. We demonstrated that tests will provide markedly better suppression of disease spread when testing is conducted closer to arrival, consistent with previous studies [11–13] and across a vast range of scenarios. Highly accurate pre-arrival testing, when conducted at an optimal time, is the least restrictive and perhaps the most promising of strategies in reducing post-arrival transmission. Our results from a broad range of scenarios and parameterization illustrate that rapid antigen tests could serve as a suitable and at times better alternative to RT-PCR, particularly if it takes 12 h or longer to receive test results.

It has been reported that rapid antigen tests alone are not sufficient in effectively identifying COVID-19 cases, because they have lower diagnostic sensitivities than RT-PCR [35]. In high-risk settings especially, RT-PCR continues to be the gold standard, and rapid antigen tests are suggested as adjunctive to other diagnostic tools [36]. However, the practical implications of when, where, and at what cost a test can be performed is a crucial component to their utility. Some countries require RT-PCR testing prior to travel and will not accept results from rapid antigen tests [5]. Pre-departure RT-PCR tests are sometimes coupled with another RT-PCR test upon entry, or additional quarantine regimens are applied, depending on the caseload in the country of origin. Specifying full adherence to self-isolation and testing protocols, we find that a rapid antigen test on the day of arrival would aid in the detection and isolation of cases, being more effective than RT-PCR testing—with turnaround time of 12 h or more—in reducing post-arrival transmission. This result is consistent with empirical evidence that rapid antigen testing 3 days prior to arrival can be effective [37], but reveals that testing closer to the day of arrival will significantly reduce transmission at events or after travel.

The trials to determine the sensitivity of most rapid antigen tests were conducted under optimal conditions, which are not necessarily representative of their practical real-world implementation. Test performance may differ substantially under practical conditions, and in the context of travel or events, may lead to post-arrival transmission values divergent from what we observed in this analysis. Independent academic investigations into the relative sensitivity for each rapid antigen test across the disease time course in a practical real-world implementation would enable increasingly accurate quantification of transmission. In a comparison of these real-world tests to those in a controlled clinical setting, a previous study demonstrated that there was similar effectiveness under these different conditions [23]. Therefore, we expect the effectiveness of each rapid antigen test presented here to be consistent in real-world implementation.

The quantification of the probability of post-arrival transmission provides a measure of effectiveness of a control strategy that accounts for the average number of secondary cases generated after arrival and individual variation in the secondary cases, but this measure is strongly dependent on the effective reproduction number. Using a basic reproductive number *R*_0_ of 6.93 ^26,27^ (quantifying the expected number of cases generated by each case) and a negative-binomially distributed number of cases generated by each case with a dispersion parameter of 0.25 [30, 31], the probability of post-arrival transmission for testing at arrival across the rapid antigen tests considered ranged from 32% to 34% compared to 35% for an RT-PCR test conducted 12 h prior to arrival. The effective reproductive number varies depending on many factors, such as the proportion of the population susceptible, behavioral variation, and public health interventions in place. For example, the probability of post-arrival transmission will change with the evolution of the epidemic, increases in vaccination uptake, or adjustments in the enforcement/practice of disease control measures. New variants of SARS-CoV-2 have been substantially increasing the transmissibility of the virus and its effective reproduction number. Our quantification of transmission depending on the day of test is entirely dependent on the baseline level of transmission, which can differ from location to location because of differences in contact patterns, population demographics, and socio-economic status. However, because the expected post-arrival transmission scales linearly with baseline values for *R*, the relative utility of the tests across days remains unchanged with changes in the effective reproductive number, which changes with time over the course of the epidemic. Thus, the relative benefit of testing at times prior to travel or events will be consistent for any levels of vaccination coverage or immunity in the population, both of which scale *R*. In the context of the dispersion parameter, the differences between the probability of post-arrival transmission become magnified for larger dispersion parameter values, favoring the use of rapid antigen test over RT-PCR. At smaller and smaller values of the dispersion parameter, the probabilities of post-arrival transmission become increasingly similar across the different tests, diminishing the impact of the choice of test on outbreak suppression. Overall, our results comparing the probability of post-arrival transmission for rapid antigen test to RT-PCR testing to highlight the benefits of rapid turnaround and testing close to arrival are qualitatively robust.

The diagnostic sensitivity of RT-PCR and rapid antigen tests have been suggested to be lower for asymptomatic cases than symptomatic cases. However, empirical studies have found that the differences in transmission between symptomatic and asymptomatic cases are moderate, especially during the initial stages of infection [17, 38–40]. This apparent reduced sensitivity among asymptomatic cases relative to symptomatic cases can be related to the time of testing over the disease time course. In the clinical trials for rapid antigen tests, samples of symptomatic individuals were concentrated around the periods of high viral loads at which symptoms appeared. In contrast, asymptomatic individuals were typically sampled across the entire disease course. As a result of this long sampling period and an unknown time of infection, the average percent positive agreement for asymptomatic cases was on average lower, appearing as a lower diagnostic sensitivity. Thus, the reduction in diagnostic sensitivity for asymptomatic cases relative to symptomatic cases might be attributable to evaluation of the tests at distinct distributions of times across the disease course [41].

A number of previous analyses have assumed a constant diagnostic sensitivity instead of a temporally varying diagnostic sensitivity for COVID-19 tests [42–44]. However, this assumption implies an equal probability of detecting infection over the course of disease, even at times when the virus is not detectable. There is not yet a strong consensus on the initial sensitivity of RT-PCR tests and changes in sensitivity over the disease course. The temporal components of the average diagnostic sensitivity of a test arise due to the individual and temporal variation in viral dynamics relative to the clinical sensitivity of the test. Many studies have demonstrated a decline in viral load as the disease progresses [19, 38, 40, 45, 46], subsequently leading to a decline in RT-PCR diagnostic sensitivity [8, 24, 31, 47, 48]. While initial sensitivity for RT-PCR can be over 85%, several studies have reported significantly lower diagnostic performance [8, 49, 50]. Further, there is little agreement on the extent and exact timing of the decline in RT-PCR diagnostic sensitivity [8, 47, 48]. Confusion regarding the temporal sensitivity of RT-PCR testing can challenge achieving informative conclusions about its relative utility compared to antigen testing [51]. Rapid antigen tests detect active viral replication and produce fewer false-negatives when an individual is most infectious than in the extremely early stages or later stages of disease when detection of antigen is difficult. These temporal changes in the average diagnostic sensitivity during the early stages of disease carry greater influence on the extent of post-arrival transmission than those later in the disease time course. For example, we estimated that the diagnostic sensitivity of the Ellume rapid antigen test rapidly dropped after 12 days of exhibiting symptoms (Supplementary Figure S12). Yet, the Ellume rapid antigen had similar effectiveness in reducing post-arrival transmission as RT-PCR (Supplementary Tables S1–S6). Thus, the ability to detect a case prior to or at times of high infectivity is critical to mitigating post-arrival transmission.

Travel can rapidly disseminate disease and new variants across the globe, and large gatherings without sufficient vaccination, boosting, and testing can lead to surges in incidence [52–54]. The identification of cases before entry to a new, populous locale or to social mixing can be a key to the prevention of rising incidence from introduced variants of concern and super-spreading, provided pre-arrival testing is conducted as close to arrival as possible.
