# Supplementary material for: Testing for COVID-19 is Much More Effective When Performed Immediately Prior to Social Mixing
Source: Int J Public Health. 2022 Jul 27;67:1604659. doi: 10.3389/ijph.2022.1604659 (PMC9363582; doi:10.3389/ijph.2022.1604659)
Supplement: Supplementary file 1 [file DataSheet1.PDF]

Supplementary Material: Testing for COVID-19 is much more effective when performed immediately prior to social mixing

Chad R. Wells[1], Senay Gokcebel[2,3], Abhishek Pandey[1], Alison P. Galvani[1], and Jeffrey P. Townsend[4, 5, 6,*]

[1] Center for Infectious Disease Modeling and Analysis (CIDMA), Yale School of Public Health, New Haven, Connecticut 06520, USA

[2]Yale School of Public Health, New Haven, Connecticut 06510, USA

[3]Grinnell College, Grinnell, Iowa 50112, USA

[4] Department of Biostatistics, Yale School of Public Health, New Haven, Connecticut 06510, USA

[5] Program in Computational Biology and Bioinformatics, Yale University, New Haven, Connecticut 06511, USA

[6] Program in Microbiology, Yale University, New Haven, Connecticut 06511, USA

*Corresponding author: Jeffrey.Townsend@yale.edu

Supplementary Figure

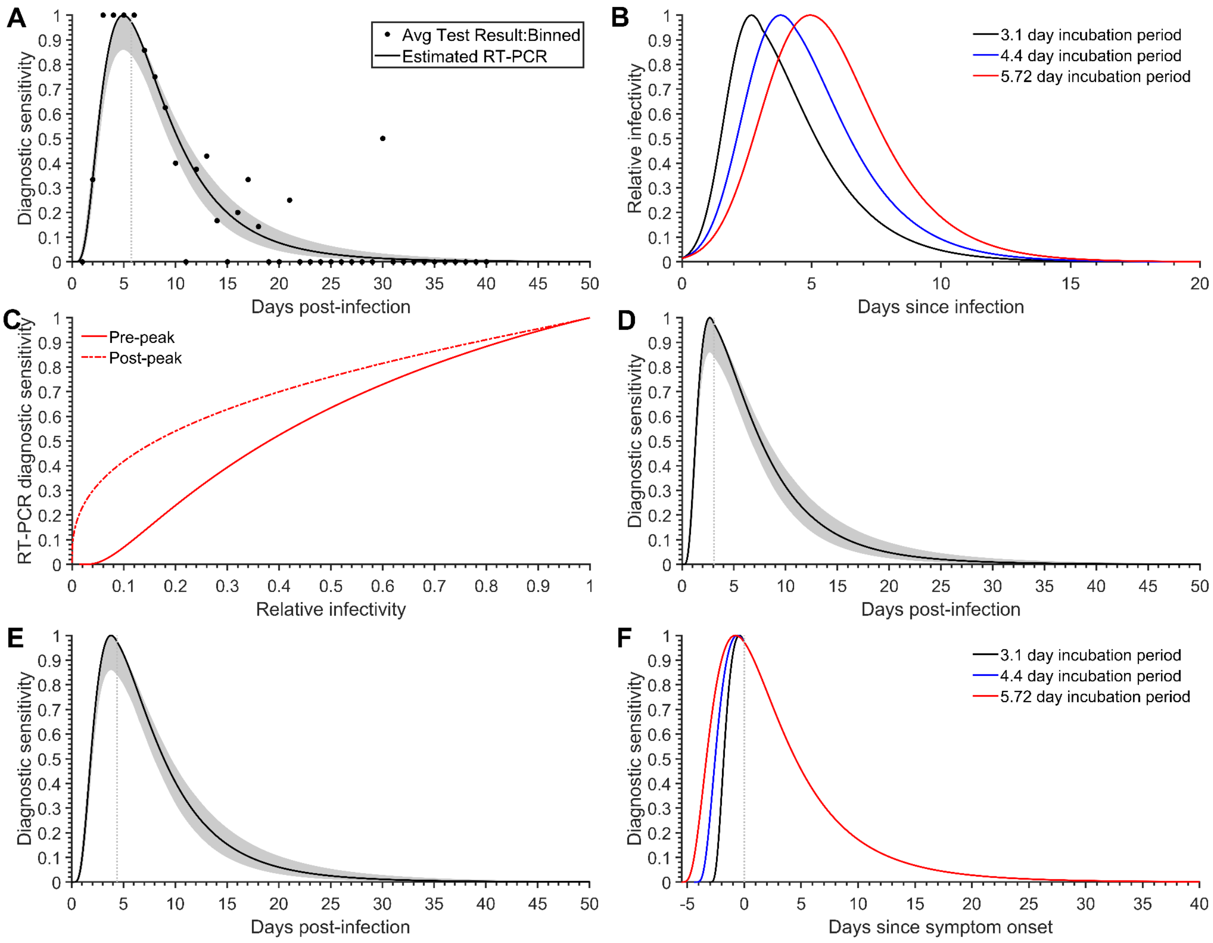

Supplementary Figure 1: RT-PCR diagnostic sensitivity over the disease time course. (**A**) The RT-PCR diagnostic sensitivity curve with baseline parameterization (solid line) and 95% credible interval (shaded area), for incubation period of 5.72 days, including the corresponding original SARS CoV-2 strain empirical data from Hellewell et al [1] (black dots; binned proportion of positive RT-PCR tests based on the inferred time of infection). (**B**)The relative infectivity (i.e. infectivity profile divided by the magnitude of peak infectivity) for the baseline 3.1-day (black), the 4.4-day (blue), and 5.72-day (red) incubation periods. (**C**) The piecewise mapping from the relative infectivity pre- and post-peak infectivity to the RT-PCR diagnostic sensitivity determined from the 5.72 day incubation period. The RT-PCR diagnostic sensitivity curve with baseline parameterization (solid line) and 95% credible interval (shaded area), for incubation periods of (**D**) 3.1 days and (**E**) 4.4 days. (**F**) A comparison of the diagnostic sensitivity curves for the baseline 3.1-day (black), the 4.4-day (blue), and 5.72-day (red) incubation periods relative to the time of symptom onset. (United States, 2021–2022)

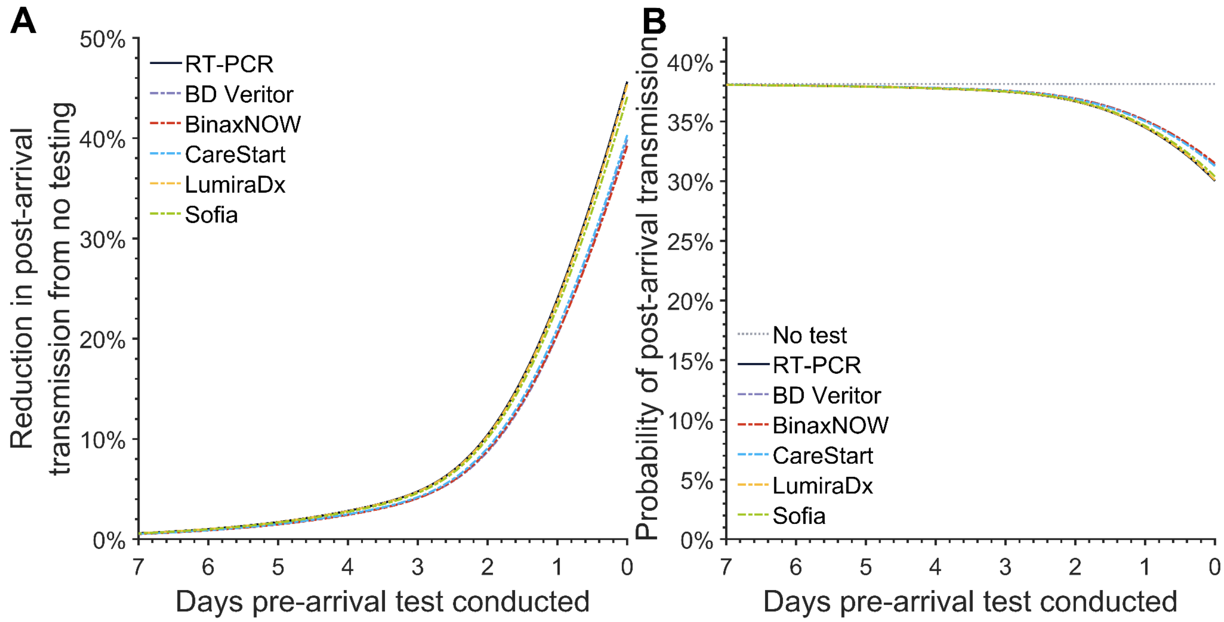

Supplementary Figure 2: The post-arrival transmission for pre-arrival testing. Specifying 35.1% infections being asymptomatic, an incubation period of 4.4 days, an RT-PCR diagnostic sensitivity curve based on the Hellewell et al [1] data, we calculated (**A**) the reduction in the post-arrival transmission compared to no pre-departure testing and (**B**) the probability of post-arrival transmission for a basic reproduction number of 5.08 when there is no pre-departure testing (gray dotted line), or when there is pre-departure testing using an RT-PCR test (black solid line), the BD Veritor (purple dashed line), BinaxNOW (red dashed line), CareStart (blue dashed line), LumiraDx (yellow dashed line), and Sofia (green dashed line) rapid antigen test conducted at departure or any previous day, up to seven days prior to departure. (United States, 2021–2022)

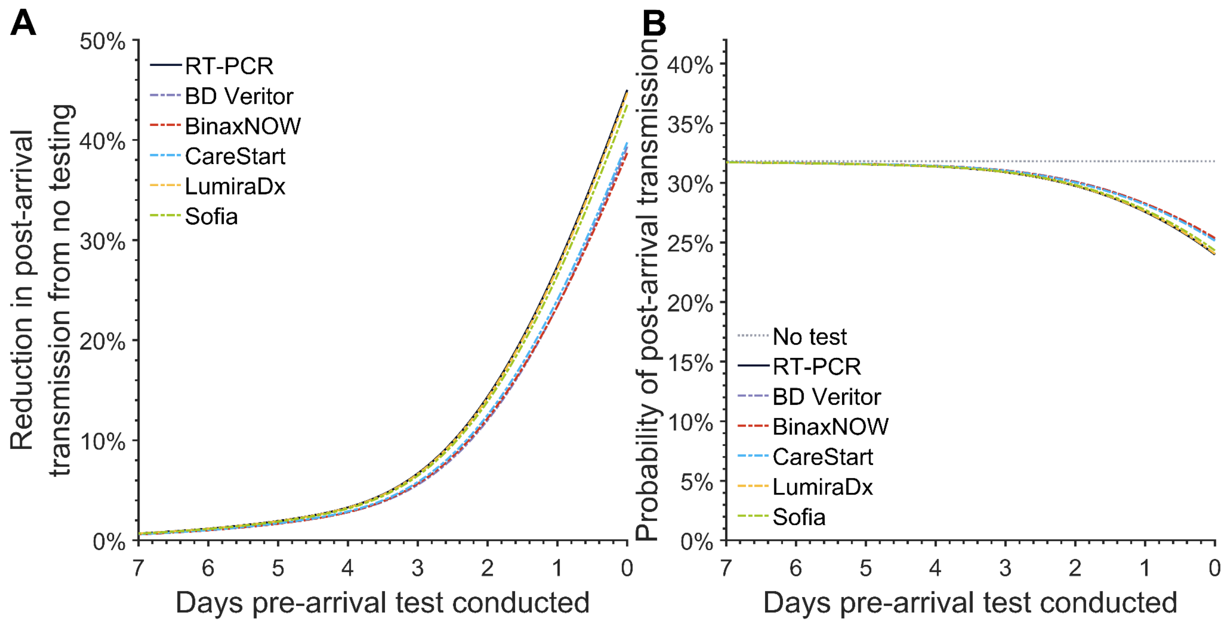

Supplementary Figure 3: The post-arrival transmission for pre-arrival testing. Specifying 35.1% infections being asymptomatic, an incubation period of 5.72 days, an RT-PCR diagnostic sensitivity curve based on the Hellewell et al [1] data, we calculated (**A**) the reduction in the post-arrival transmission compared to no pre-departure testing and (**B**) the probability of post-arrival transmission for a basic reproduction number of 2.79 when there is no pre-departure testing (gray dotted line), or when there is pre-departure testing using an RT-PCR test (black solid line), the BD Veritor (purple dashed line), BinaxNOW (red dashed line), CareStart (blue dashed line), LumiraDx (yellow dashed line), and Sofia (green dashed line) rapid antigen test conducted at departure or any previous day, up to seven days prior to departure. (United States, 2021–2022)

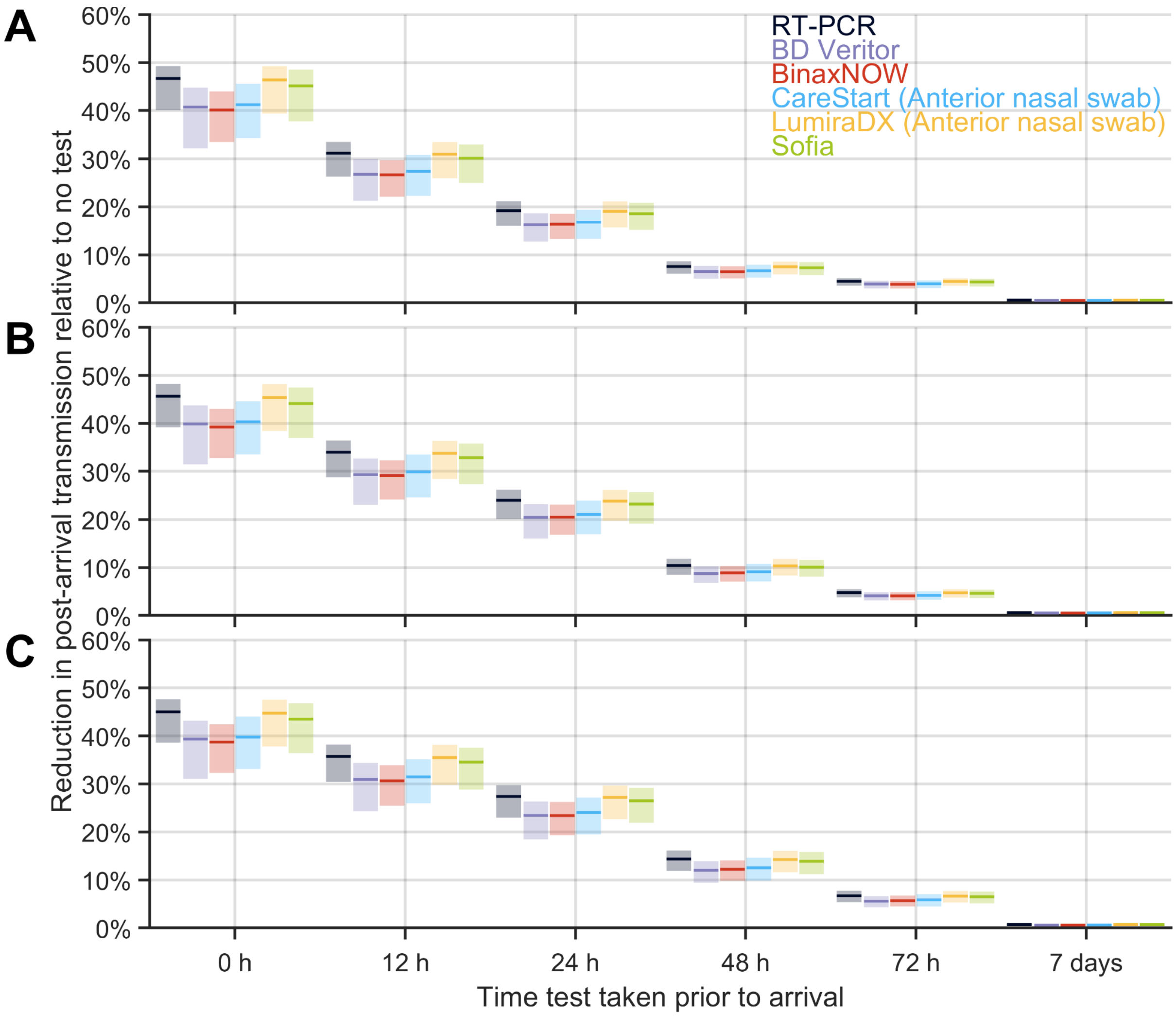

Supplementary Figure 4: Uncertainty in the reduction of post-arrival transmission for pre-arrival testing. Specifying 35.1% (95% CrI: 30.6%–39.8%) infections being asymptomatic and an RT-PCR diagnostic sensitivity curve based on the Hellewell et al [1] data, we calculated the expected post-arrival transmission at the time of departure, 12 h, 24 h, 48 h, 72 h and one week prior to departure for using an RT-PCR test (black), the BD Veritor (purple), BinaxNOW (red), CareStart (blue), LumiraDx (yellow), and Sofia (green) rapid antigen test in a scenario of a (**A**) 3.1 day incubation period, a (**B**) 4.4 day incubation period, and a (**C**) 5.72 day incubation period. The shaded region denotes the 95% credible interval based on 1,000 samples of the parameters determining the diagnostic sensitivity and the proportion of infections that are asymptomatic. (United States, 2021–2022)

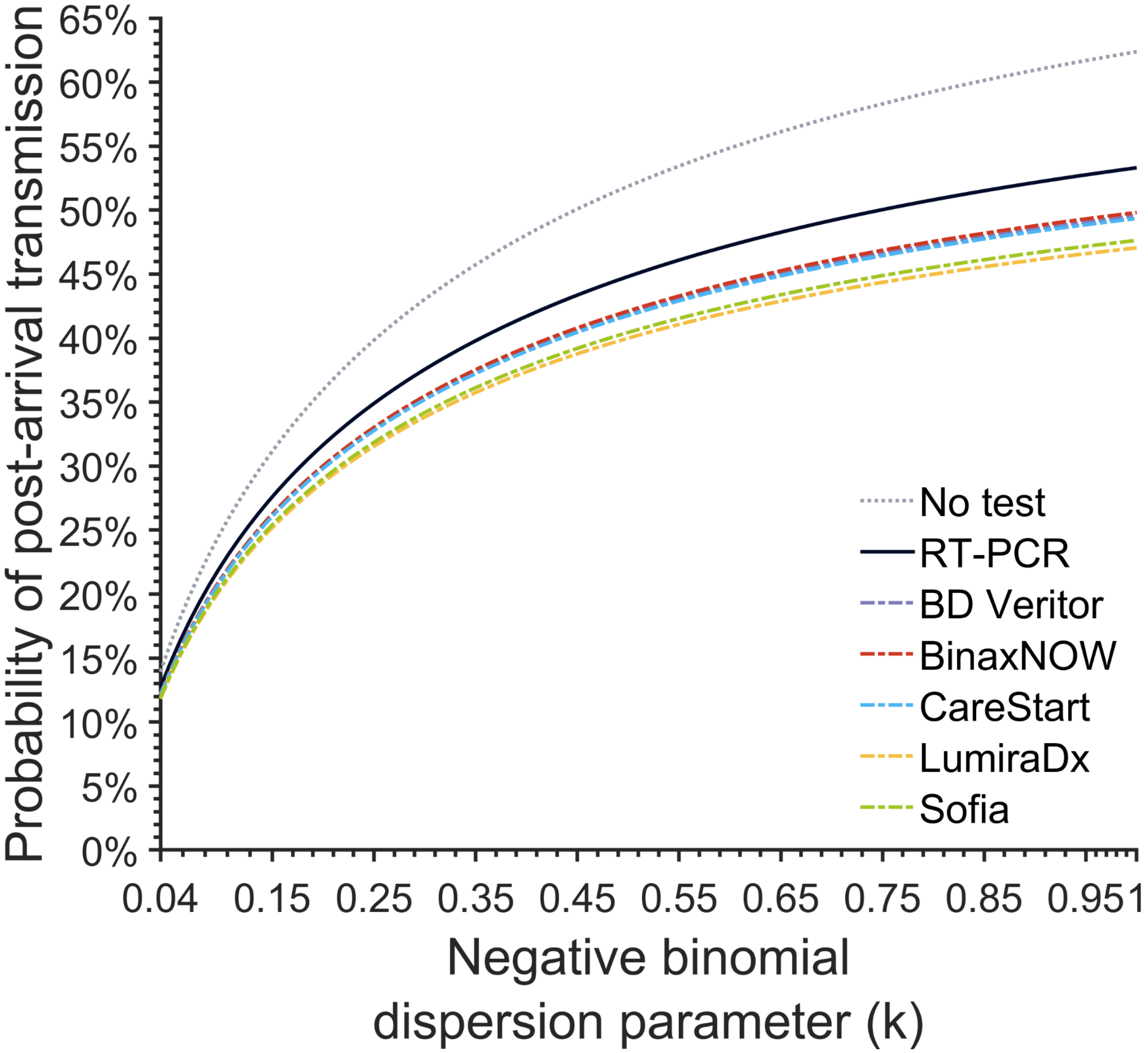

Supplementary Figure 5: The post-arrival transmission for pre-arrival testing for different negative binomial dispersion parameter values. Specifying 35.1% infections being asymptomatic, an incubation period of 3.1 days, an RT-PCR diagnostic sensitivity curve based on the Hellewell et al [1] data, we calculated the probability of post-arrival transmission for a basic reproduction number of 6.93 when there is no pre-departure testing (gray dotted line), when there is pre-departure testing 12-h prior to arrival using an RT-PCR test (black solid line), or when there is testing upon arrival using the BD Veritor (purple dashed line), BinaxNOW (red dashed line), CareStart (blue dashed line), LumiraDx (yellow dashed line), and Sofia (green dashed line) rapid antigen test conducted for negative binomial dispersion parameter values *k* between 0.04 and 1. (United States, 2021–2022)

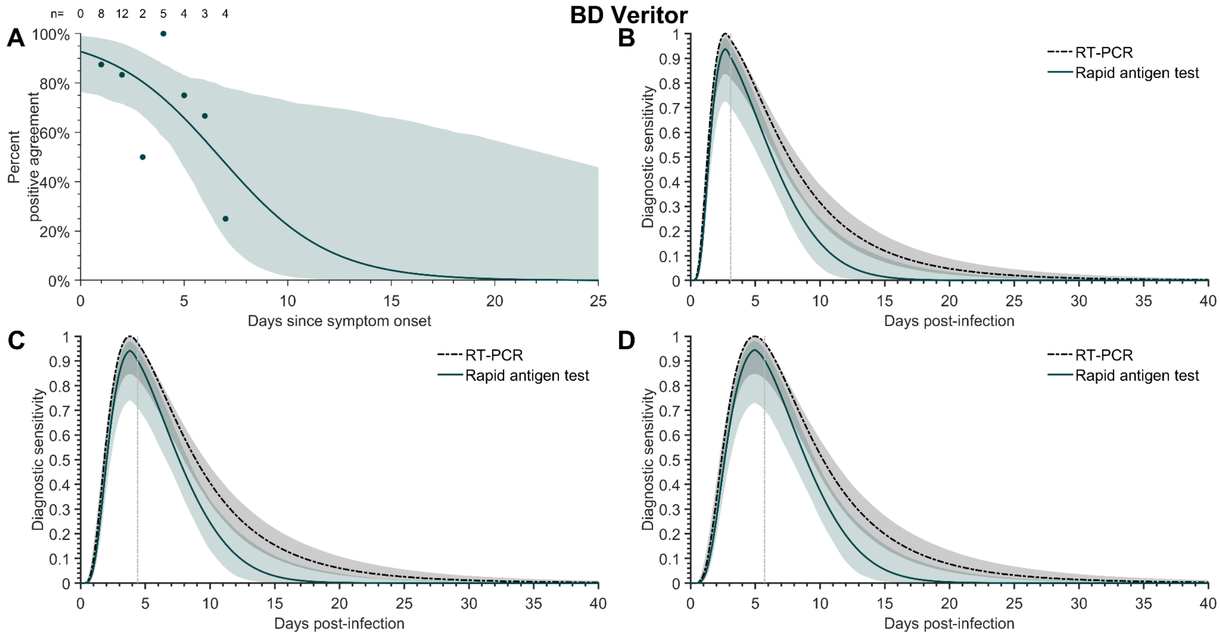

Supplementary Figure 6: The percent positive agreement of BD Veritor with RT-PCR and corresponding diagnostic sensitivity. Using a linear logit-model to determine the percent positive agreement of a rapid antigen test, (**A**) the fitted linear-logit model (green line) and 95% credible interval (green-shaded region), and the United States Food and Drug Administration reported percent positive agreement (open circles: percent positive agreement data spanning multiple days; closed circles: percent positive agreement data spanning multiple days) of the rapid antigen test with RT-PCR from the time of symptom onset. The diagnostic sensitivity of the rapid antigen test (green line) and 95% credible interval (green shaded area) compared to the diagnostic sensitivity of RT-PCR (black line) and 95% credible interval (black shaded area) when specifying (**B**) a 3.1-day, (**C**) a 4.4-day, and (**D**) a 5.72-day incubation period. Uncertainty in the estimates was quantified using 1,000 samples drawn by likelihood-weighted importance sampling. (United States, 2021–2022)

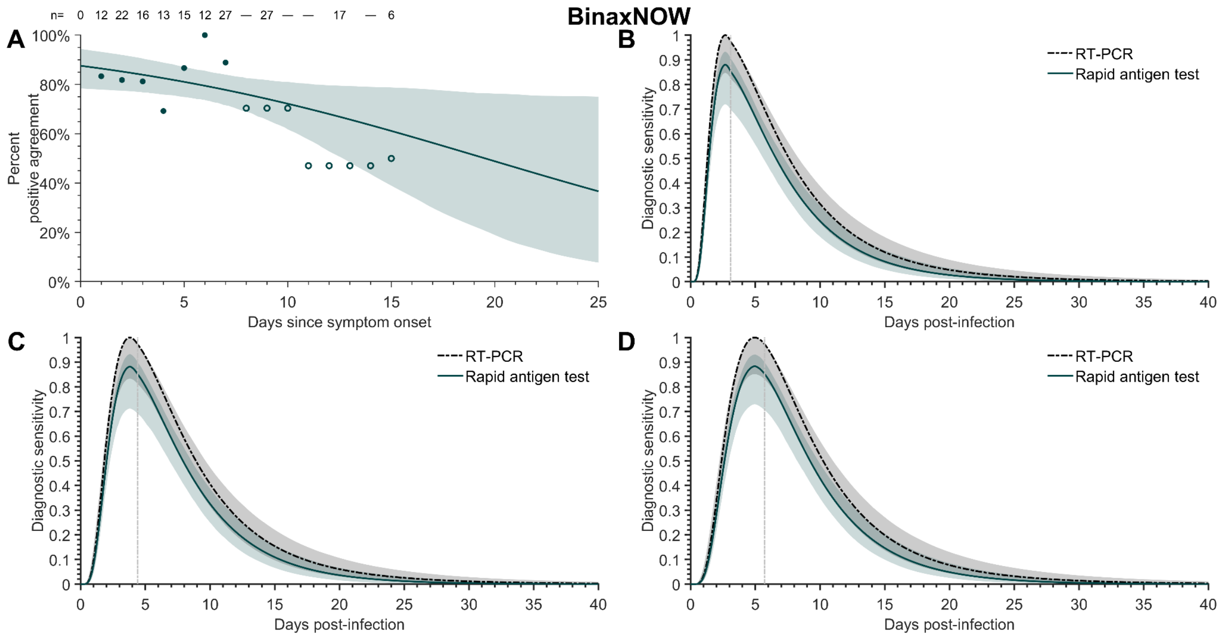

Supplementary Figure 7: The percent positive agreement of BinaxNOW with RT-PCR and corresponding diagnostic sensitivity. Using a linear logit-model to determine the percent positive agreement of a rapid antigen test, (**A**) the fitted linear-logit model (green line) and 95% credible interval (green-shaded region), and the United States Food and Drug Administration reported percent positive agreement (open circles: percent positive agreement data spanning multiple days; closed circles: percent positive agreement data spanning multiple days) of the rapid antigen test with RT-PCR from the time of symptom onset. The diagnostic sensitivity of the rapid antigen test (green line) and 95% credible interval (green shaded area) compared to the diagnostic sensitivity of RT-PCR (black line) and 95% credible interval (black shaded area) when specifying (**B**) a 3.1-day, (**C**) a 4.4-day, and (**D**) a 5.72-day incubation period. Uncertainty in the estimates was quantified using 1,000 samples drawn by likelihood-weighted importance sampling. (United States, 2021–2022)

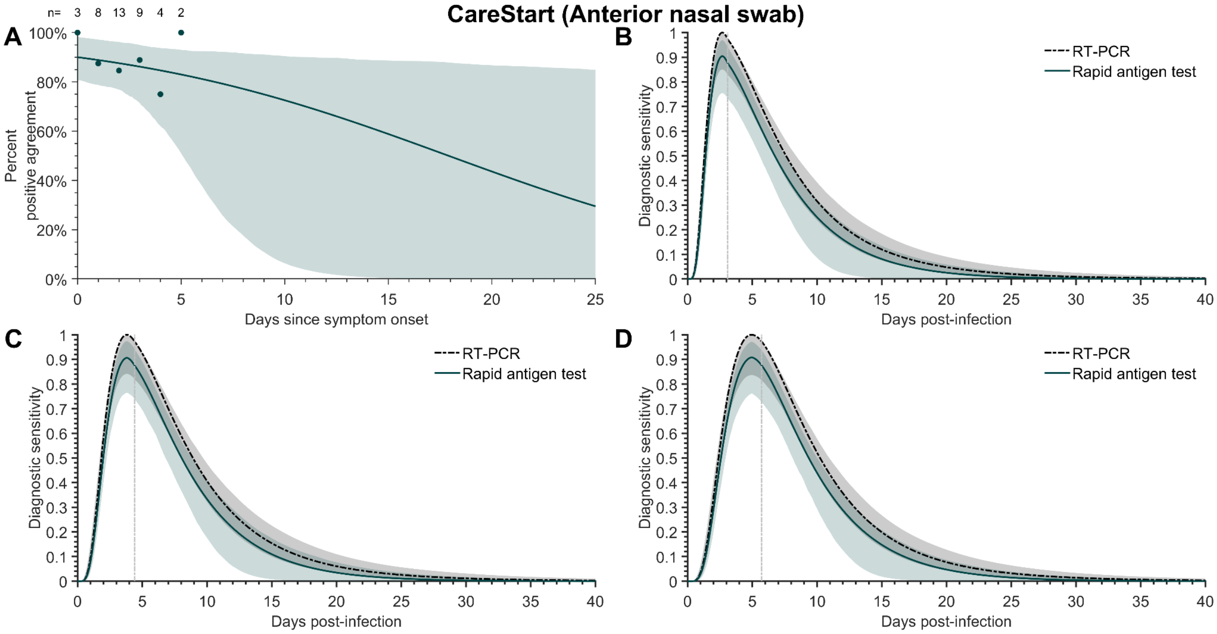

Supplementary Figure 8: The percent positive agreement of CareStart anterior nasal swab with RT-PCR and corresponding diagnostic sensitivity. Using a linear logit-model to determine the percent positive agreement of a rapid antigen test, (**A**) the fitted linear-logit model (green line) and 95% credible interval (green-shaded region), and the United States Food and Drug Administration reported percent positive agreement (open circles: percent positive agreement data spanning multiple days; closed circles: percent positive agreement data spanning multiple days) of the rapid antigen test with RT-PCR from the time of symptom onset. The diagnostic sensitivity of the rapid antigen test (green line) and 95% credible interval (green shaded area) compared to the diagnostic sensitivity of RT-PCR (black line) and 95% credible interval (black shaded area) when specifying (**B**) a 3.1-day, (**C**) a 4.4-day, and (**D**) a 5.72-day incubation period. Uncertainty in the estimates was quantified using 1,000 samples drawn by likelihood-weighted importance sampling. (United States, 2021–2022)

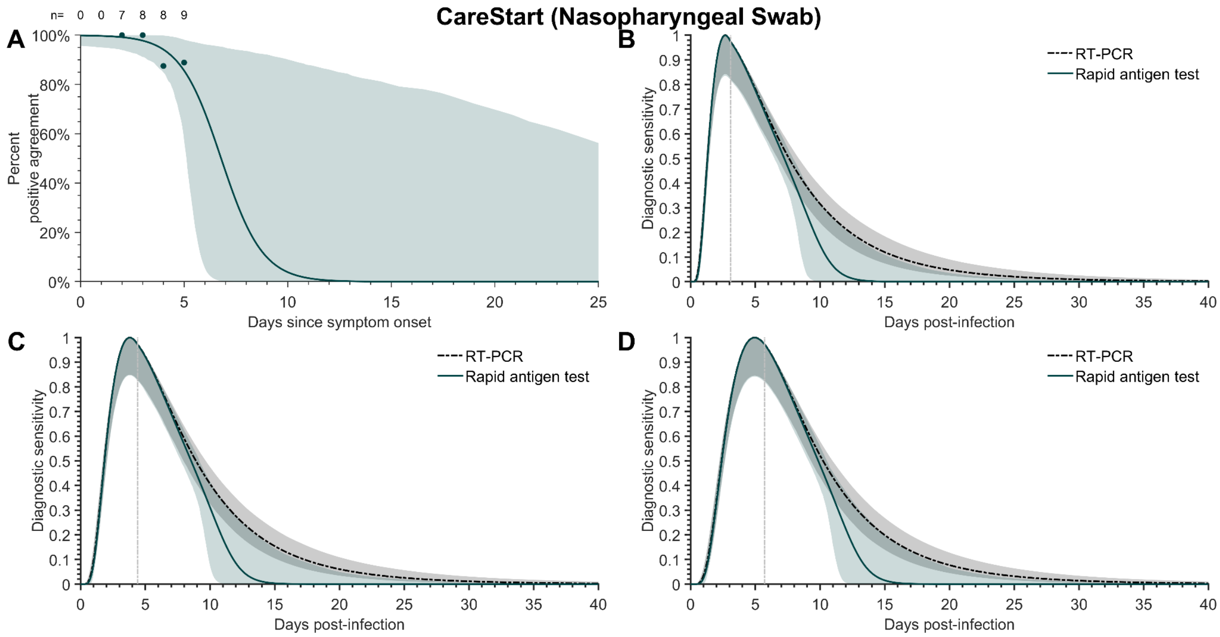

Supplementary Figure 9: The percent positive agreement of CareStart nasopharyngeal swab with RT-PCR and corresponding diagnostic sensitivity. Using a linear logit-model to determine the percent positive agreement of a rapid antigen test, (**A**) the fitted linear-logit model (green line) and 95% credible interval (green-shaded region), and the United States Food and Drug Administration reported percent positive agreement (open circles: percent positive agreement data spanning multiple days; closed circles: percent positive agreement data spanning multiple days) of the rapid antigen test with RT-PCR from the time of symptom onset. The diagnostic sensitivity of the rapid antigen test (green line) and 95% credible interval (green shaded area) compared to the diagnostic sensitivity of RT-PCR (black line) and 95% credible interval (black shaded area) when specifying (**B**) a 3.1-day, (**C**) a 4.4-day, and (D) a 5.72-day incubation period. Uncertainty in the estimates was quantified using 1,000 samples drawn by likelihood-weighted importance sampling. (United States, 2021–2022)

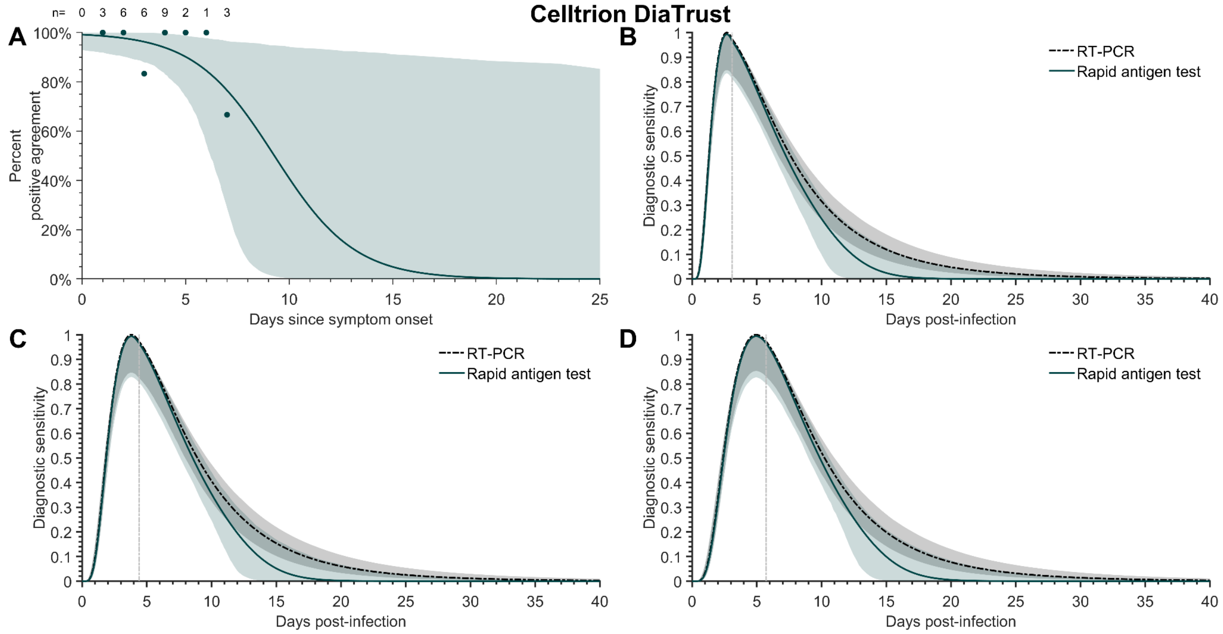

Supplementary Figure 10: The percent positive agreement of Celltrion Dia Trust with RT-PCR and corresponding diagnostic sensitivity. Using a linear logit-model to determine the percent positive agreement of a rapid antigen test, (**A**) the fitted linear-logit model (green line) and 95% credible interval (green-shaded region), and the United States Food and Drug Administration reported percent positive agreement (open circles: percent positive agreement data spanning multiple days; closed circles: percent positive agreement data spanning multiple days) of the rapid antigen test with RT-PCR from the time of symptom onset. The diagnostic sensitivity of the rapid antigen test (green line) and 95% credible interval (green shaded area) compared to the diagnostic sensitivity of RT-PCR (black line) and 95% credible interval (black shaded area) when specifying (**B**) a 3.1-day, (**C**) a 4.4-day, and (**D**) a 5.72-day incubation period. Uncertainty in the estimates was quantified using 1,000 samples drawn by likelihood-weighted importance sampling. (United States, 2021–2022)

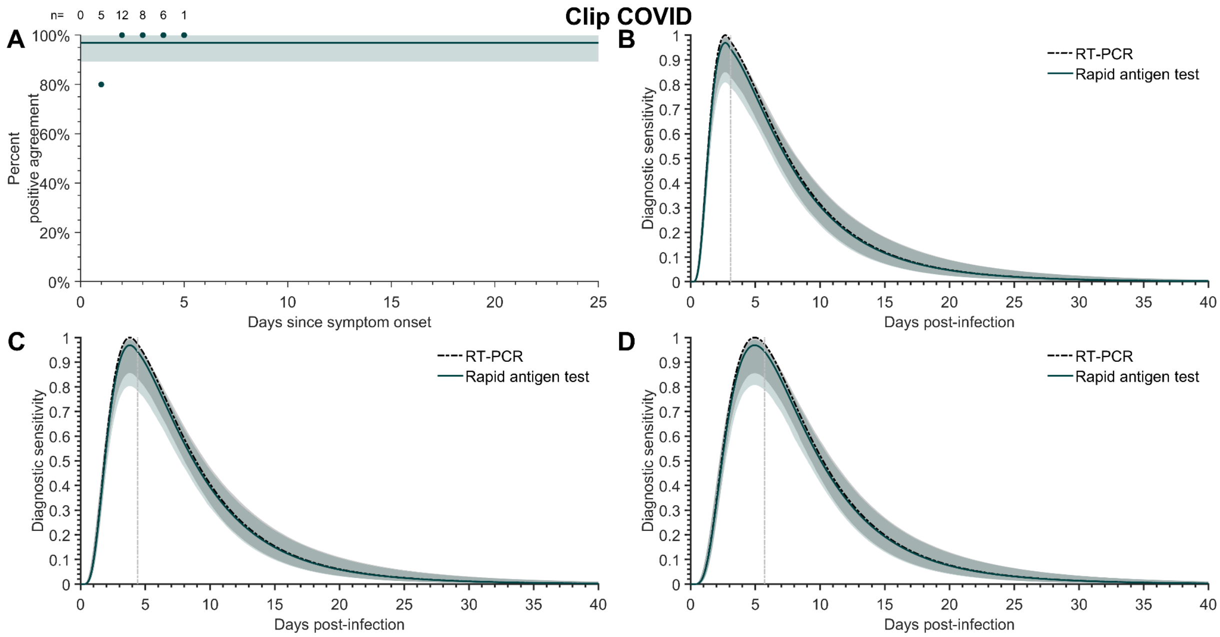

Supplementary Figure 11: The percent positive agreement of Clip COVID with RT-PCR and corresponding diagnostic sensitivity. Using a linear logit-model to determine the percent positive agreement of a rapid antigen test, (**A**) the fitted linear-logit model (green line) and 95% credible interval (green-shaded region), and the United States Food and Drug Administration reported percent positive agreement (open circles: percent positive agreement data spanning multiple days; closed circles: percent positive agreement data spanning multiple days) of the rapid antigen test with RT-PCR from the time of symptom onset. The diagnostic sensitivity of the rapid antigen test (green line) and 95% credible interval (green shaded area) compared to the diagnostic sensitivity of RT-PCR (black line) and 95% credible interval (black shaded area) when specifying (**B**) a 3.1-day, (**C**) a 4.4-day, and (**D**) a 5.72-day incubation period. Uncertainty in the estimates was quantified using 1,000 samples drawn by likelihood-weighted importance sampling. (United States, 2021–2022)

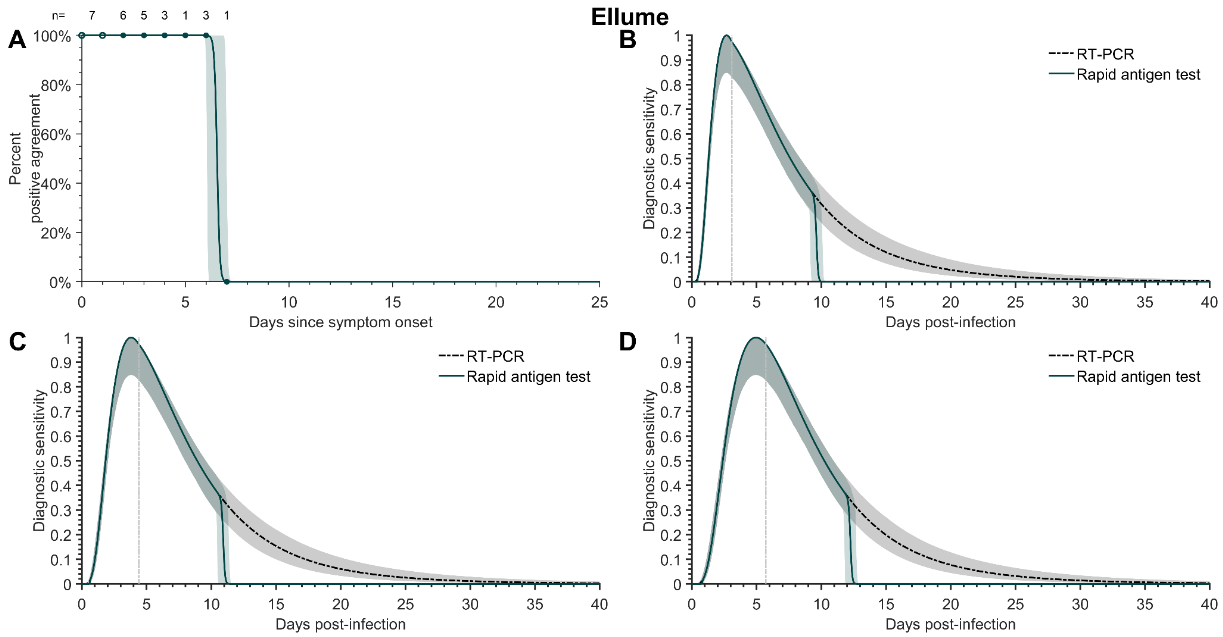

Supplementary Figure 12: The percent positive agreement of Ellume with RT-PCR and corresponding diagnostic sensitivity. Using a linear logit-model to determine the percent positive agreement of a rapid antigen test, (**A**) the fitted linear-logit model (green line) and 95% credible interval (green-shaded region), and the United States Food and Drug Administration reported percent positive agreement (open circles: percent positive agreement data spanning multiple days; closed circles: percent positive agreement data spanning multiple days) of the rapid antigen test with RT-PCR from the time of symptom onset. The diagnostic sensitivity of the rapid antigen test (green line) and 95% credible interval (green shaded area) compared to the diagnostic sensitivity of RT-PCR (black line) and 95% credible interval (black shaded area) when specifying (**B**) a 3.1-day, (**C**) a 4.4-day, and (**D**) a 5.72-day incubation period. Uncertainty in the estimates was quantified using 1,000 samples drawn by likelihood-weighted importance sampling. (United States, 2021–2022)

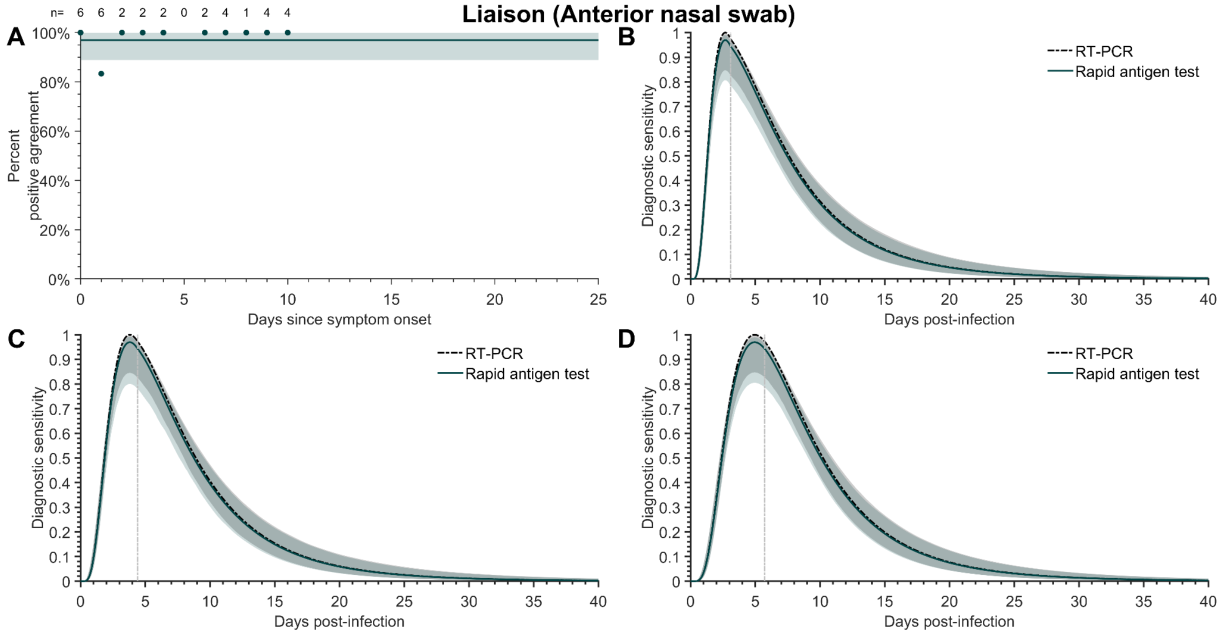

Supplementary Figure 13: The percent positive agreement of Liaison anterior nasal swab with RT-PCR and corresponding diagnostic sensitivity. Using a linear logit-model to determine the percent positive agreement of a rapid antigen test, (**A**) the fitted linear-logit model (green line) and 95% credible interval (green-shaded region), and the United States Food and Drug Administration reported percent positive agreement (open circles: percent positive agreement data spanning multiple days; closed circles: percent positive agreement data spanning multiple days) of the rapid antigen test with RT-PCR from the time of symptom onset. The diagnostic sensitivity of the rapid antigen test (green line) and 95% credible interval (green shaded area) compared to the diagnostic sensitivity of RT-PCR (black line) and 95% credible interval (black shaded area) when specifying (**B**) a 3.1-day, (**C**) a 4.4-day, and (**D**) a 5.72-day incubation period. Uncertainty in the estimates was quantified using 1,000 samples drawn by likelihood-weighted importance sampling. (United States, 2021–2022)

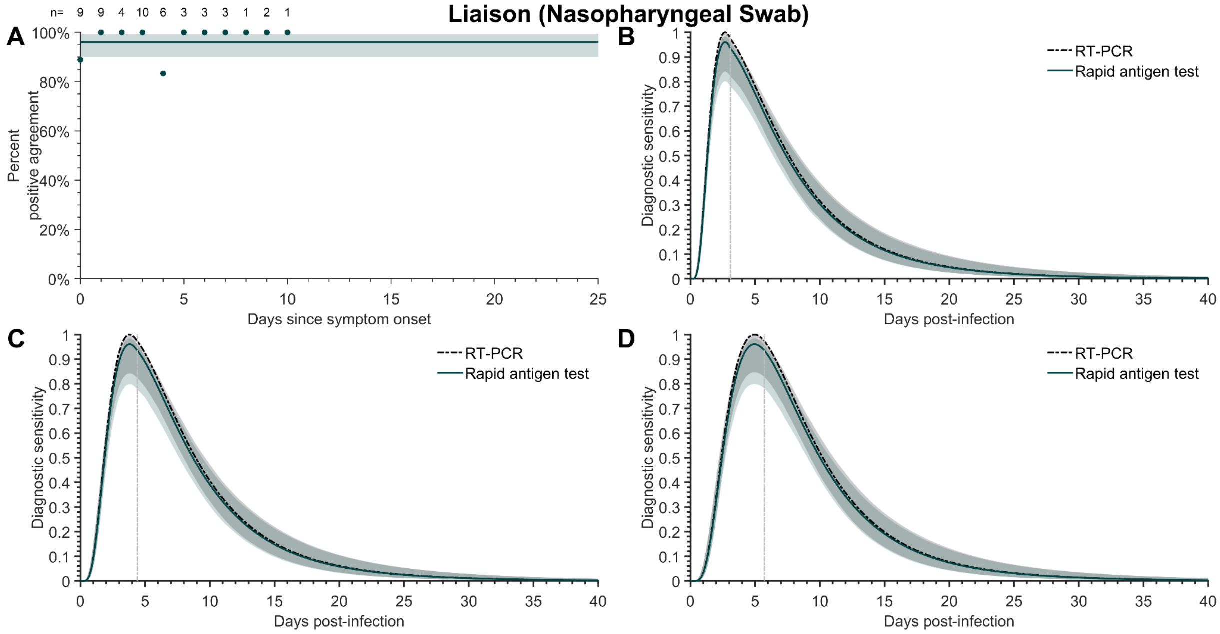

Supplementary Figure 14: The percent positive agreement of Liaison nasopharyngeal swab with RT-PCR and corresponding diagnostic sensitivity. Using a linear logit-model to determine the percent positive agreement of a rapid antigen test, (**A**) the fitted linear-logit model (green line) and 95% credible interval (green-shaded region), and the United States Food and Drug Administration reported percent positive agreement (open circles: percent positive agreement data spanning multiple days; closed circles: percent positive agreement data spanning multiple days) of the rapid antigen test with RT-PCR from the time of symptom onset. The diagnostic sensitivity of the rapid antigen test (green line) and 95% credible interval (green shaded area) compared to the diagnostic sensitivity of RT-PCR (black line) and 95% credible interval (black shaded area) when specifying (**B**) a 3.1-day, (**C**) a 4.4-day, and (**D**) a 5.72-day incubation period. Uncertainty in the estimates was quantified using 1,000 samples drawn by likelihood-weighted importance sampling. (United States, 2021–2022)

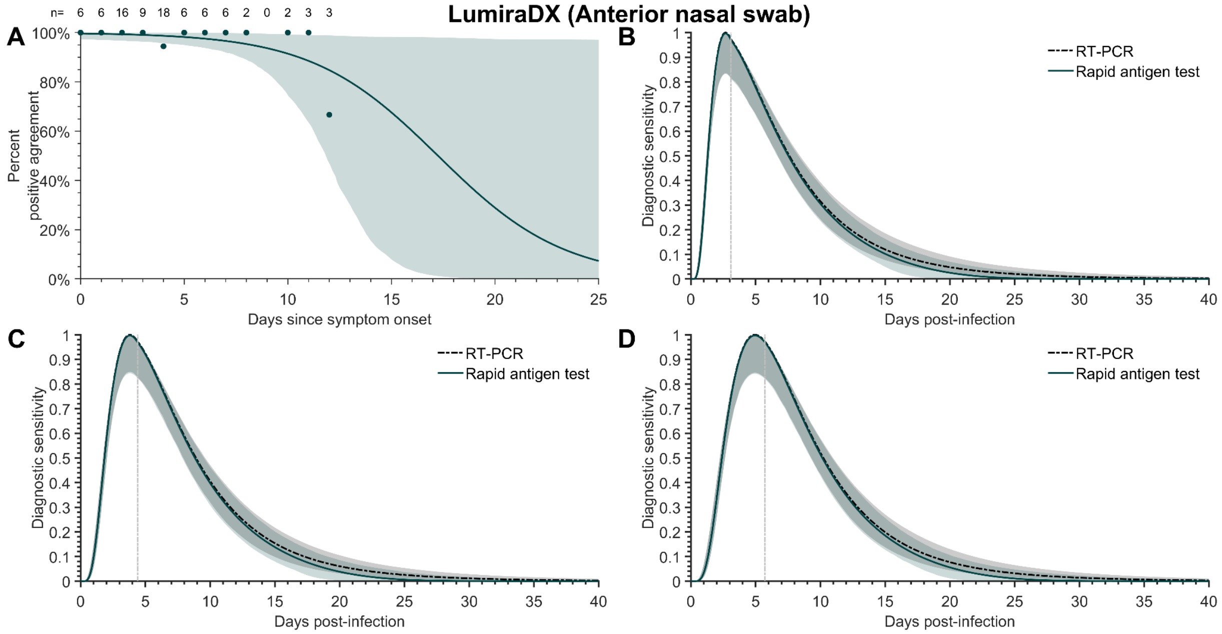

Supplementary Figure 15: The percent positive agreement of LumiraDx anterior nasal swab with RT-PCR and corresponding diagnostic sensitivity. Using a linear logit-model to determine the percent positive agreement of a rapid antigen test, (**A**) the fitted linear-logit model (green line) and 95% credible interval (green-shaded region), and the United States Food and Drug Administration reported percent positive agreement (open circles: percent positive agreement data spanning multiple days; closed circles: percent positive agreement data spanning multiple days) of the rapid antigen test with RT-PCR from the time of symptom onset. The diagnostic sensitivity of the rapid antigen test (green line) and 95% credible interval (green shaded area) compared to the diagnostic sensitivity of RT-PCR (black line) and 95% credible interval (black shaded area) when specifying (**B**) a 3.1-day, (**C**) a 4.4-day, and (**D**) a 5.72-day incubation period. Uncertainty in the estimates was quantified using 1,000 samples drawn by likelihood-weighted importance sampling. (United States, 2021–2022)

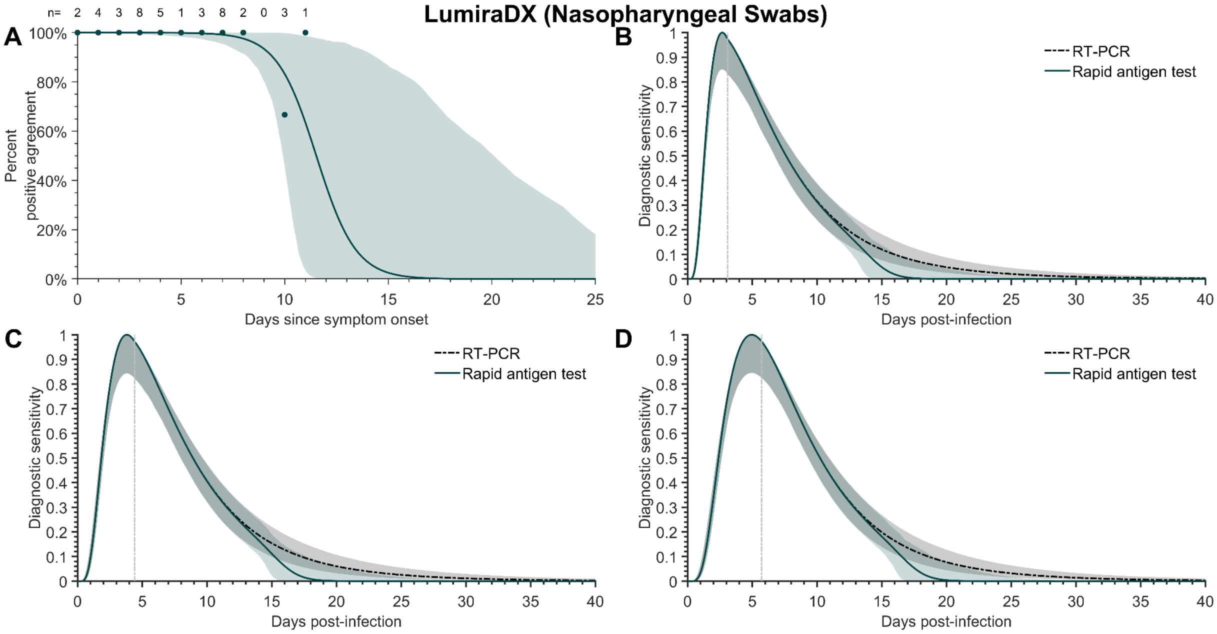

Supplementary Figure 16: The percent positive agreement of LumiraDX nasopharyngeal swab with RT-PCR and corresponding diagnostic sensitivity. Using a linear logit-model to determine the percent positive agreement of a rapid antigen test, (**A**) the fitted linear-logit model (green line) and 95% credible interval (green-shaded region), and the United States Food and Drug Administration reported percent positive agreement (open circles: percent positive agreement data spanning multiple days; closed circles: percent positive agreement data spanning multiple days) of the rapid antigen test with RT-PCR from the time of symptom onset. The diagnostic sensitivity of the rapid antigen test (green line) and 95% credible interval (green shaded area) compared to the diagnostic sensitivity of RT-PCR (black line) and 95% credible interval (black shaded area) when specifying (**B**) a 3.1-day, (**C**) a 4.4-day, and (**D**) a 5.72-day incubation period. Uncertainty in the estimates was quantified using 1,000 samples drawn by likelihood-weighted importance sampling. (United States, 2021–2022)

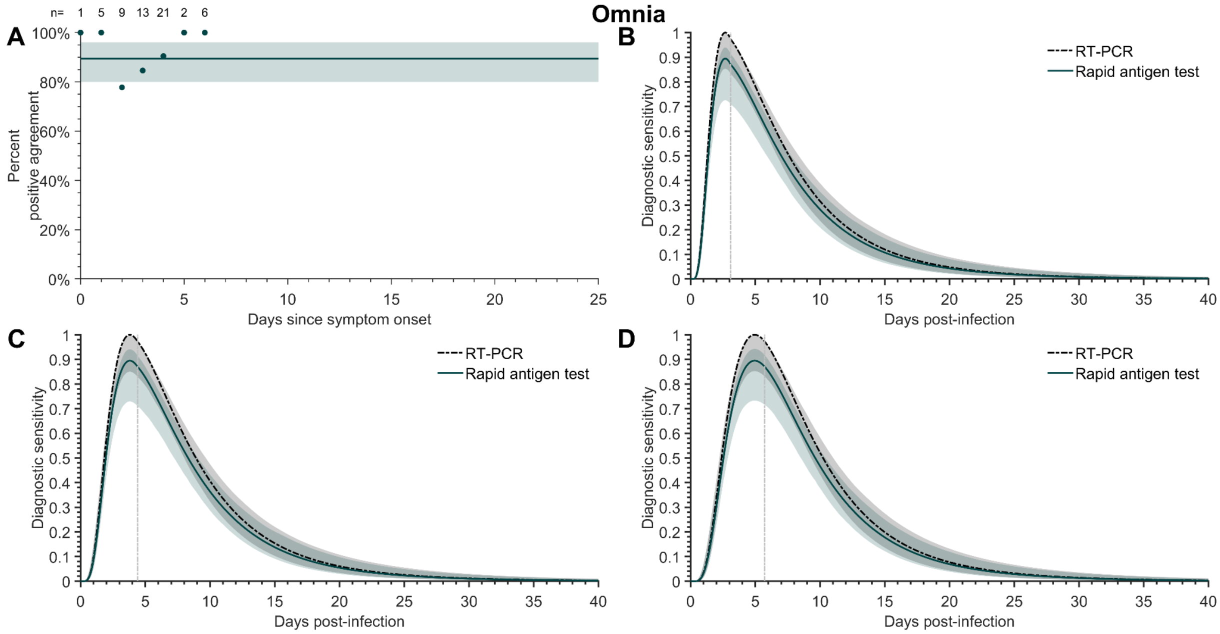

Supplementary Figure 17: The percent positive agreement of Omnia with RT-PCR and corresponding diagnostic sensitivity. Using a linear logit-model to determine the percent positive agreement of a rapid antigen test, (**A**) the fitted linear-logit model (green line) and 95% credible interval (green-shaded region), and the United States Food and Drug Administration reported percent positive agreement (open circles: percent positive agreement data spanning multiple days; closed circles: percent positive agreement data spanning multiple days) of the rapid antigen test with RT-PCR from the time of symptom onset. The diagnostic sensitivity of the rapid antigen test (green line) and 95% credible interval (green shaded area) compared to the diagnostic sensitivity of RT-PCR (black line) and 95% credible interval (black shaded area) when specifying (**B**) a 3.1-day, (**C**) a 4.4-day, and (**D**) a 5.72-day incubation period. Uncertainty in the estimates was quantified using 1,000 samples drawn by likelihood-weighted importance sampling. (United States, 2021–2022)

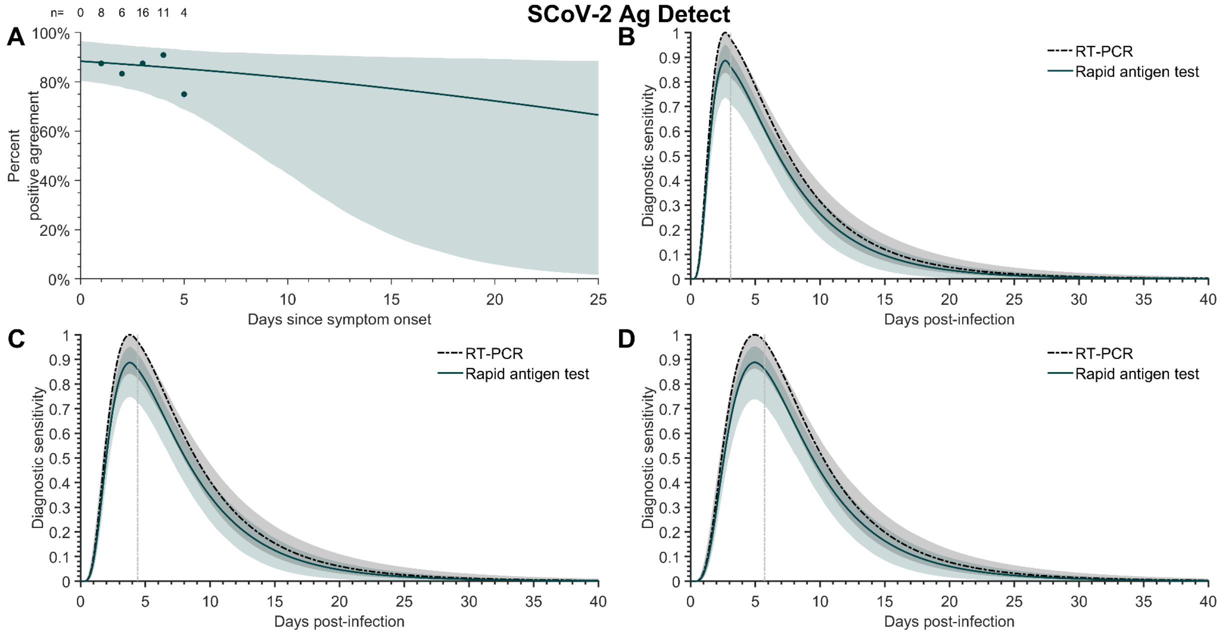

Supplementary Figure 18: The percent positive agreement of SCoV-2 Ag Detect with RT-PCR and corresponding diagnostic sensitivity. Using a linear logit-model to determine the percent positive agreement of a rapid antigen test, (**A**) the fitted linear-logit model (green line) and 95% credible interval (green-shaded region), and the United States Food and Drug Administration reported percent positive agreement (open circles: percent positive agreement data spanning multiple days; closed circles: percent positive agreement data spanning multiple days) of the rapid antigen test with RT-PCR from the time of symptom onset. The diagnostic sensitivity of the rapid antigen test (green line) and 95% credible interval (green shaded area) compared to the diagnostic sensitivity of RT-PCR (black line) and 95% credible interval (black shaded area) when specifying (**B**) a 3.1-day, (**C**) a 4.4-day, and (**D**) a 5.72-day incubation period. Uncertainty in the estimates was quantified using 1,000 samples drawn by likelihood-weighted importance sampling. (United States, 2021–2022)

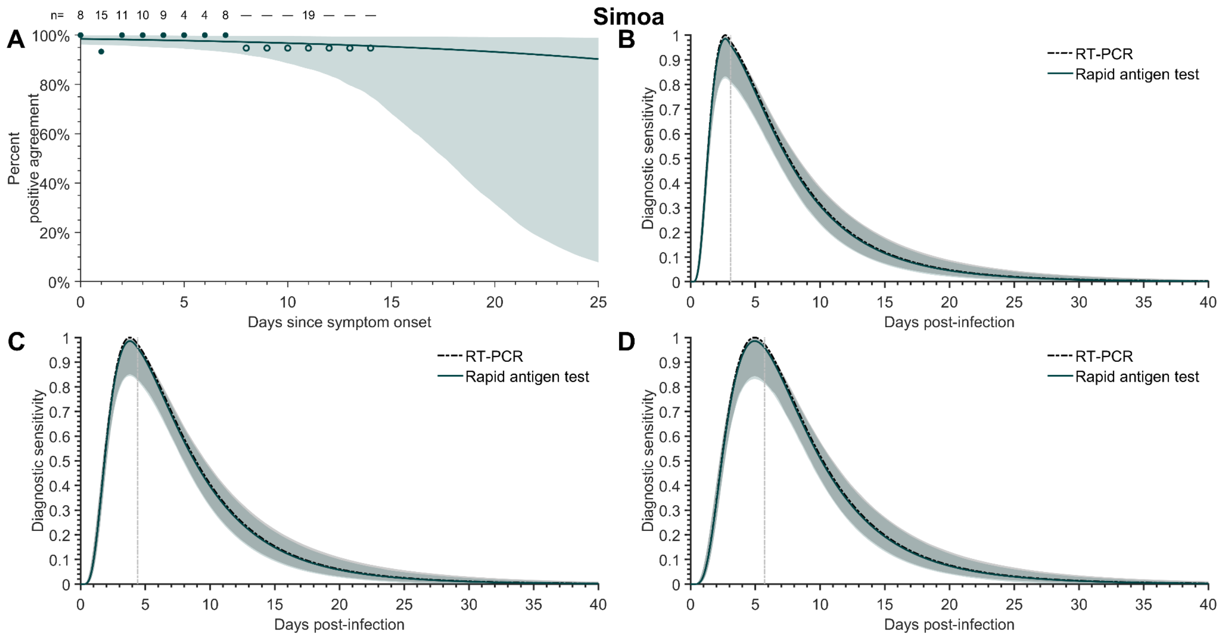

Supplementary Figure 19: The percent positive agreement of Simoa with RT-PCR and corresponding diagnostic sensitivity. Using a linear logit-model to determine the percent positive agreement of a rapid antigen test, (**A**) the fitted linear-logit model (green line) and 95% credible interval (green-shaded region), and the United States Food and Drug Administration reported percent positive agreement (open circles: percent positive agreement data spanning multiple days; closed circles: percent positive agreement data spanning multiple days) of the rapid antigen test with RT-PCR from the time of symptom onset. The diagnostic sensitivity of the rapid antigen test (green line) and 95% credible interval (green shaded area) compared to the diagnostic sensitivity of RT-PCR (black line) and 95% credible interval (black shaded area) when specifying (**B**) a 3.1-day, (**C**) a 4.4-day, and (**D**) a 5.72-day incubation period. Uncertainty in the estimates was quantified using 1,000 samples drawn by likelihood-weighted importance sampling. (United States, 2021–2022)

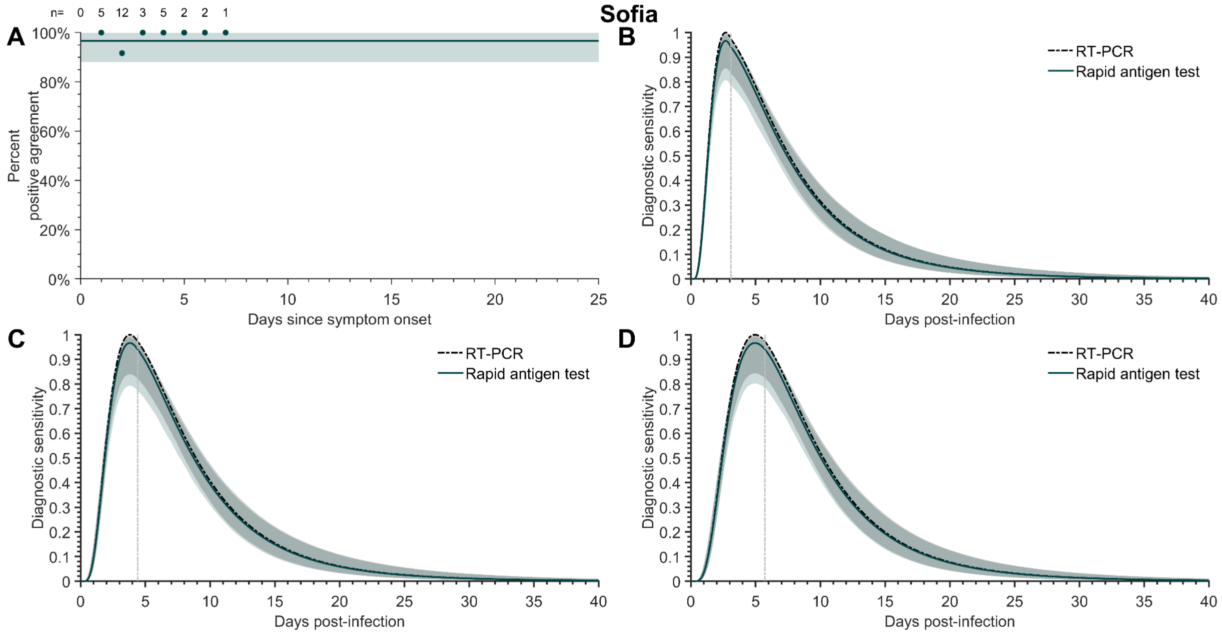

Supplementary Figure 20: The percent positive agreement of Sofia with RT-PCR and corresponding diagnostic sensitivity. Using a linear logit-model to determine the percent positive agreement of a rapid antigen test, (**A**) the fitted linear-logit model (green line) and 95% credible interval (green-shaded region), and the United States Food and Drug Administration reported percent positive agreement (open circles: percent positive agreement data spanning multiple days; closed circles: percent positive agreement data spanning multiple days) of the rapid antigen test with RT-PCR from the time of symptom onset. The diagnostic sensitivity of the rapid antigen test (green line) and 95% credible interval (green shaded area) compared to the diagnostic sensitivity of RT-PCR (black line) and 95% credible interval (black shaded area) when specifying (**B**) a 3.1-day, (**C**) a 4.4-day, and (**D**) a 5.72-day incubation period. Uncertainty in the estimates was quantified using 1,000 samples drawn by likelihood-weighted importance sampling. (United States, 2021–2022)

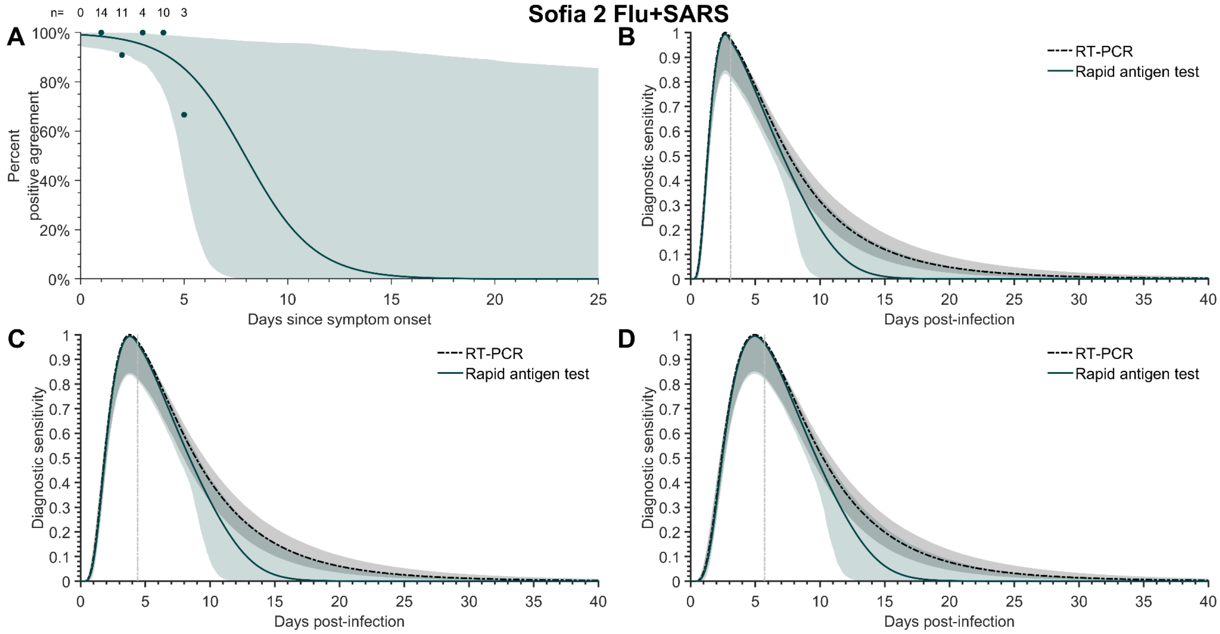

Supplementary Figure 21: The percent positive agreement of Sofia 2 Flu+SARS with RT-PCR and corresponding diagnostic sensitivity. Using a linear logit-model to determine the percent positive agreement of a rapid antigen test, (**A**) the fitted linear-logit model (green line) and 95% credible interval (green-shaded region), and the United States Food and Drug Administration reported percent positive agreement (open circles: percent positive agreement data spanning multiple days; closed circles: percent positive agreement data spanning multiple days) of the rapid antigen test with RT-PCR from the time of symptom onset. The diagnostic sensitivity of the rapid antigen test (green line) and 95% credible interval (green shaded area) compared to the diagnostic sensitivity of RT-PCR (black line) and 95% credible interval (black shaded area) when specifying (**B**) a 3.1-day, (**C**) a 4.4-day, and (**D**) a 5.72-day incubation period. Uncertainty in the estimates was quantified using 1,000 samples drawn by likelihood-weighted importance sampling. (United States, 2021–2022)

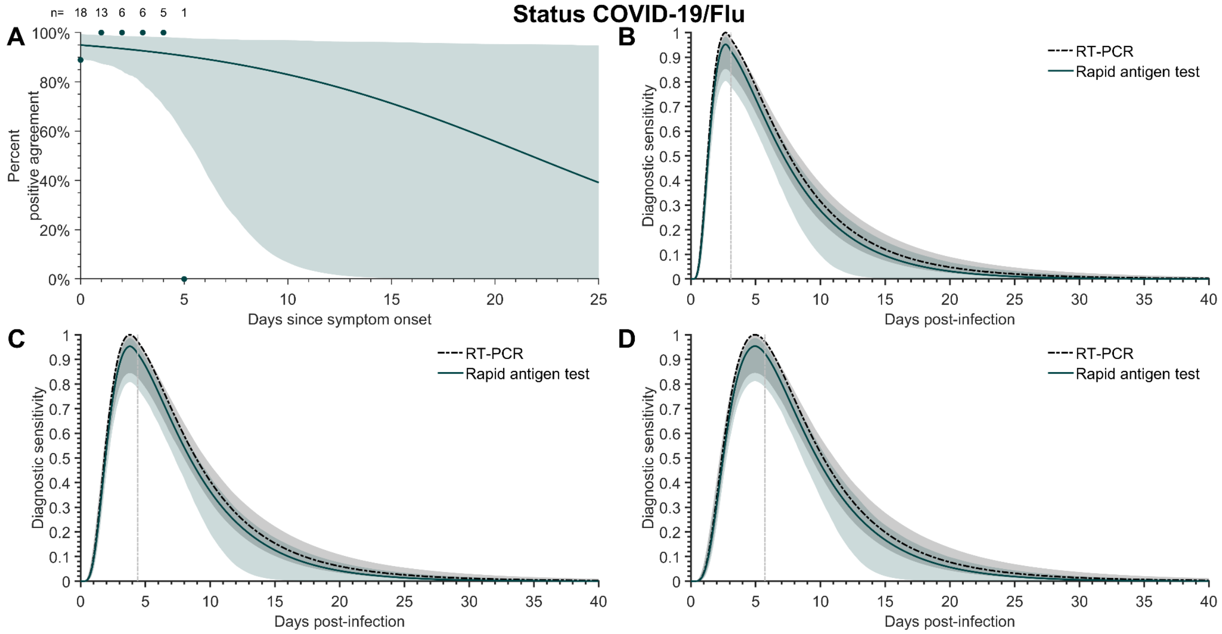

Supplementary Figure 22: The percent positive agreement of Status COVID-19/Flu with RT-PCR and corresponding diagnostic sensitivity. Using a linear logit-model to determine the percent positive agreement of a rapid antigen test, (**A**) the fitted linear-logit model (green line) and 95% credible interval (green-shaded region), and the United States Food and Drug Administration reported percent positive agreement (open circles: percent positive agreement data spanning multiple days; closed circles: percent positive agreement data spanning multiple days) of the rapid antigen test with RT-PCR from the time of symptom onset. The diagnostic sensitivity of the rapid antigen test (green line) and 95% credible interval (green shaded area) compared to the diagnostic sensitivity of RT-PCR (black line) and 95% credible interval (black shaded area) when specifying (**B**) a 3.1-day, (**C**) a 4.4-day, and (**D**) a 5.72-day incubation period. Uncertainty in the estimates was quantified using 1,000 samples drawn by likelihood-weighted importance sampling. (United States, 2021–2022)

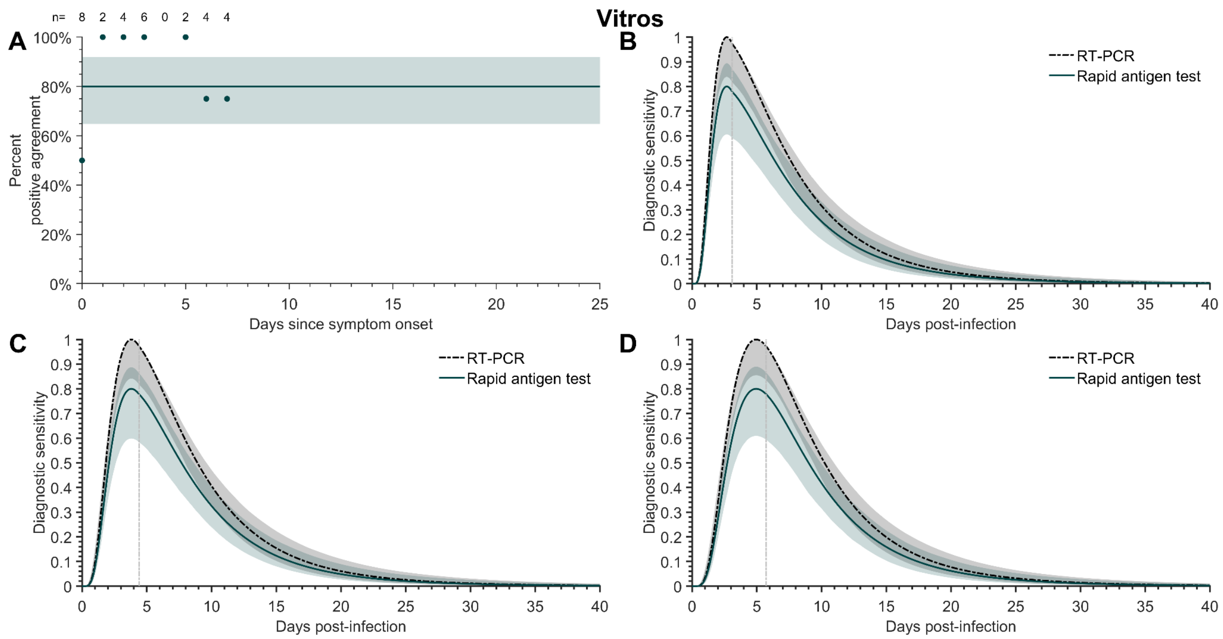

Supplementary Figure 23: The percent positive agreement of Vitros with RT-PCR and corresponding diagnostic sensitivity. Using a linear logit-model to determine the percent positive agreement of a rapid antigen test, (**A**) the fitted linear-logit model (green line) and 95% credible interval (green-shaded region), and the United States Food and Drug Administration reported percent positive agreement (open circles: percent positive agreement data spanning multiple days; closed circles: percent positive agreement data spanning multiple days) of the rapid antigen test with RT-PCR from the time of symptom onset. The diagnostic sensitivity of the rapid antigen test (green line) and 95% credible interval (green shaded area) compared to the diagnostic sensitivity of RT-PCR (black line) and 95% credible interval (black shaded area) when specifying (**B**) a 3.1-day, (**C**) a 4.4-day, and (**D**) a 5.72-day incubation period. Uncertainty in the estimates was quantified using 1,000 samples drawn by likelihood-weighted importance sampling. (United States, 2021–2022)

Supplementary Tables

Supplementary Table 1: Reduction in the expected post-arrival transmission as a result of pre-arrival testing relative to no pre-arrival testing for RT-PCR, 18 rapid antigen tests, and an incubation period of 3.1 days. (United States, 2021–2022)

Test	Hours pre-departure				
	72	48	24	12	0
RT-PCR	4.5% (3.6%–5.1%)[a]	7.6% (6.1%–8.6%)	19.2% (16.0%–21.1%)	31.1% (26.3%–33.5%)	46.7% (40.1%–49.3%)
BD Veritor	3.9% (3.0%–4.6%)	6.5% (5.0%–7.7%)	16.2% (12.8%–18.6%)	26.7% (21.2%–29.9%)	40.8% (32.2%–44.8%)
BinaxNOW	3.9% (3.0%–4.5%)	6.5% (5.1%–7.6%)	16.4% (13.3%–18.5%)	26.6% (22.1%–29.7%)	40.1% (33.5%–44.0%)
CareStart [b]	4.0% (3.1%–4.7%)	6.7% (5.2%–7.9%)	16.8% (13.3%–19.3%)	27.4% (22.3%–30.8%)	41.2% (34.3%–45.6%)
CareStart [c]	4.4% (3.5%–5.0%)	7.4% (5.9%–8.4%)	18.7% (15.6%–20.6%)	30.5% (25.9%–32.8%)	46.0% (39.4%–48.5%)
Celltrion DiaTrust	4.4% (3.5%–5.0%)	7.4% (5.9%–8.3%)	18.6% (15.4%–20.6%)	30.3% (25.6%–32.8%)	45.7% (38.7%–48.4%)
Clip COVID	4.3% (3.4%–5.0%)	7.3% (5.8%–8.4%)	18.6% (15.2%–20.7%)	30.2% (25.0%–32.8%)	45.2% (37.8%–48.4%)
Ellume	4.5% (3.6%–5.1%)	7.5% (6.0%–8.5%)	19.1% (15.9%–21.0%)	31.0% (26.2%–33.3%)	46.6% (40.0%–49.1%)
Liaison [b]	4.3% (3.4%–5.0%)	7.3% (5.8%–8.4%)	18.6% (15.2%–20.7%)	30.2% (25.1%–32.8%)	45.3% (37.9%–48.5%)
Liaison [c]	4.3% (3.4%–4.9%)	7.3% (5.7%–8.3%)	18.4% (15.2%–20.5%)	29.9% (25.1%–32.6%)	44.9% (37.9%–48.0%)
LumiraDX [b]	4.5% (3.5%–5.1%)	7.5% (5.9%–8.6%)	19.0% (15.7%–21.1%)	30.9% (25.9%–33.5%)	46.4% (39.4%–49.2%)
LumiraDX [c]	4.5% (3.6%–5.1%)	7.6% (6.0%–8.6%)	19.2% (15.9%–21.1%)	31.1% (26.2%–33.5%)	46.7% (39.9%–49.3%)
Omnia	4.0% (3.1%–4.6%)	6.8% (5.3%–7.8%)	17.2% (13.9%–19.3%)	27.9% (22.8%–30.7%)	41.8% (34.5%–45.3%)
SCoV-2 Ag Detect	3.9%	6.6%	16.7%	27.2%	40.9%

	(3.1%–4.6%)	(5.2%–7.8%)	(13.6%–19.2%)	(22.6%–30.4%)	(34.1%–45.1%)
Simoa	4.4%<br>(3.5%–5.1%)	7.4%<br>(6.0%–8.5%)	18.8%<br>(15.8%–20.9%)	30.6%<br>(25.9%–33.1%)	45.9%<br>(39.4%–48.8%)
Sofia	4.3%<br>(3.4%–5.0%)	7.3%<br>(5.8%–8.5%)	18.5%<br>(15.2%–20.8%)	30.1%<br>(25.0%–33.0%)	45.1%<br>(37.8%–48.5%)
Sofia 2 Flu+SARS	4.4%<br>(3.5%–5.0%)	7.3%<br>(5.8%–8.3%)	18.4%<br>(15.2%–20.5%)	30.1%<br>(25.2%–32.4%)	45.4%<br>(38.6%–48.1%)
Status COVID-19/Flu	4.2%<br>(3.3%–4.8%)	7.1%<br>(5.6%–8.1%)	17.9%<br>(14.7%–20.1%)	29.2%<br>(24.2%–31.9%)	43.8%<br>(37.2%–47.2%)
Vitros	3.6%<br>(2.6%–4.4%)	6.0%<br>(4.3%–7.5%)	15.3%<br>(11.4%–18.3%)	24.9%<br>(18.7%–29.0%)	37.4%<br>(28.2%–42.7%)

[a] 95% credible interval
[b] Anterior nasal swab
[c] Nasopharyngeal swab

Supplementary Table 2: The probability post-travel transmission without pre-departure testing and with pre-departure testing using RT-PCR, one of the 18 rapid antigen tests, an incubation period of 3.1 days, and a basic reproduction number of 6.93. (United States, 2021–2022)

Test	Hours pre-departure					
	No test	72	48	24	12	0
RT-PCR	39.8% (39.6%–40.1%)[a]	39.2% (38.9%–39.6%)	38.8% (38.4%–39.2%)	37.0% (36.5%–37.7%)	34.9% (34.3%–35.9%)	31.5% (30.7%–33.1%)
BD Veritor	39.8% (39.6%–40.1%)	39.3% (39.0%–39.6%)	38.9% (38.6%–39.4%)	37.5% (37.0%–38.1%)	35.7% (35.0%–36.8%)	32.9% (31.9%–34.7%)
BinaxNOW	39.8% (39.6%–40.1%)	39.3% (39.0%–39.7%)	39.0% (38.6%–39.4%)	37.5% (37.0%–38.1%)	35.7% (35.1%–36.6%)	33.0% (32.1%–34.4%)
CareStart [b]	39.8% (39.6%–40.1%)	39.3% (39.0%–39.6%)	38.9% (38.6%–39.3%)	37.4% (36.8%–38.1%)	35.6% (34.9%–36.6%)	32.8% (31.7%–34.4%)
CareStart [c]	39.8% (39.6%–40.1%)	39.2% (38.9%–39.6%)	38.8% (38.5%–39.3%)	37.1% (36.6%–37.8%)	35.0% (34.4%–36.0%)	31.7% (31.0%–33.2%)
Celltrion DiaTrust	39.8% (39.6%–40.1%)	39.2% (38.9%–39.6%)	38.8% (38.5%–39.3%)	37.1% (36.6%–37.8%)	35.1% (34.5%–36.0%)	31.7% (31.0%–33.4%)
Clip COVID	39.8% (39.6%–40.1%)	39.3% (38.9%–39.6%)	38.8% (38.5%–39.3%)	37.1% (36.6%–37.8%)	35.1% (34.4%–36.1%)	31.8% (31.0%–33.6%)
Ellume	39.8% (39.6%–40.1%)	39.2% (38.9%–39.6%)	38.8% (38.4%–39.2%)	37.0% (36.6%–37.7%)	34.9% (34.4%–35.9%)	31.5% (30.8%–33.1%)
Liaison [b]	39.8% (39.6%–40.1%)	39.3% (38.9%–39.6%)	38.8% (38.5%–39.3%)	37.1% (36.6%–37.8%)	35.1% (34.4%–36.1%)	31.8% (31.0%–33.6%)
Liaison [c]	39.8% (39.6%–40.1%)	39.3% (38.9%–39.6%)	38.8% (38.5%–39.3%)	37.2% (36.7%–37.8%)	35.1% (34.5%–36.1%)	31.9% (31.1%–33.5%)
LumiraDX [b]	39.8% (39.6%–40.1%)	39.2% (38.9%–39.6%)	38.8% (38.4%–39.2%)	37.1% (36.6%–37.7%)	34.9% (34.3%–36.0%)	31.5% (30.7%–33.2%)
LumiraDX [c]	39.8% (39.6%–40.1%)	39.2% (38.9%–39.6%)	38.8% (38.4%–39.2%)	37.0% (36.5%–37.7%)	34.9% (34.3%–35.9%)	31.5% (30.7%–33.1%)
Omnia	39.8% (39.6%–40.1%)	39.3% (39.0%–39.6%)	38.9% (38.6%–39.3%)	37.4% (36.9%–38.0%)	35.5% (34.9%–36.5%)	32.7% (31.7%–34.3%)
SCoV-2 Ag Detect	39.8% (39.6%–40.1%)	39.3% (39.0%–39.6%)	38.9% (38.6%–39.3%)	37.4% (36.9%–38.1%)	35.6% (34.9%–36.6%)	32.9% (31.8%–34.3%)
Simoa	39.8% (39.6%–40.1%)	39.2% (38.9%–39.6%)	38.8% (38.4%–39.2%)	37.1% (36.6%–37.7%)	35.0% (34.4%–36.0%)	31.7% (30.9%–33.3%)
Sofia	39.8%	39.3%	38.8%	37.1%	35.1%	31.9%

	(39.6%–40.1%)	(38.9%–39.6%)	(38.5%–39.3%)	(36.6%–37.8%)	(34.4%–36.1%)	(30.9%–33.6%)
Sofia 2 Flu+SARS	39.8% (39.6%–40.1%)	39.3% (38.9%–39.6%)	38.8% (38.5%–39.3%)	37.2% (36.7%–37.8%)	35.1% (34.5%–36.1%)	31.8% (31.1%–33.4%)
Status COVID-19/Flu	39.8% (39.6%–40.1%)	39.3% (39.0%–39.6%)	38.9% (38.5%–39.3%)	37.2% (36.7%–37.9%)	35.3% (34.7%–36.2%)	32.2% (31.3%–33.7%)
Vitros	39.8% (39.6%–40.1%)	39.4% (39.0%–39.7%)	39.0% (38.6%–39.4%)	37.6% (37.0%–38.4%)	36.1% (35.2%–37.2%)	33.6% (32.3%–35.5%)

[a] 95% credible interval
[b] Anterior nasal swab
[c] Nasopharyngeal swab

Supplementary Table 3: Reduction in the expected post-arrival transmission as a result of pre-arrival testing relative to no pre-arrival testing for RT-PCR testing, 18 rapid antigen tests and an incubation period of 4.4 days. (United States, 2021–2022)

Test	Hours pre-departure				
	72	48	24	12	0
RT-PCR	4.8% (3.8%–5.5%)[a]	10.4% (8.5%–11.8%)	24.0% (20.1%–26.2%)	34.0% (28.8%–36.4%)	45.6% (39.2%–48.2%)
BD Veritor	4.1% (3.2%–4.9%)	8.7% (6.8%–10.3%)	20.4% (16.0%–23.1%)	29.3% (23.1%–32.7%)	39.9% (31.5%–43.7%)
BinaxNOW	4.1% (3.2%–4.8%)	8.9% (7.1%–10.3%)	20.5% (16.8%–23.1%)	29.1% (24.1%–32.3%)	39.2% (32.8%–43.0%)
CareStart [b]	4.2% (3.3%–5.0%)	9.1% (7.1%–10.7%)	21.0% (16.9%–23.9%)	29.9% (24.6%–33.5%)	40.3% (33.6%–44.6%)
CareStart [c]	4.6% (3.7%–5.3%)	10.1% (8.2%–11.4%)	23.4% (19.7%–25.5%)	33.3% (28.3%–35.7%)	44.9% (38.5%–47.5%)
Celltrion DiaTrust	4.6% (3.7%–5.3%)	10.1% (8.2%–11.5%)	23.3% (19.5%–25.5%)	33.1% (28.0%–35.6%)	44.6% (37.9%–47.3%)
Clip COVID	4.6% (3.7%–5.4%)	10.1% (8.1%–11.5%)	23.2% (19.2%–25.6%)	32.9% (27.4%–35.7%)	44.2% (37.0%–47.4%)
Ellume	4.7% (3.8%–5.4%)	10.4% (8.5%–11.7%)	23.9% (20.0%–26.1%)	33.9% (28.7%–36.3%)	45.5% (39.1%–48.0%)
Liaison [b]	4.6% (3.7%–5.4%)	10.1% (8.1%–11.6%)	23.3% (19.3%–25.6%)	32.9% (27.5%–35.7%)	44.3% (37.1%–47.5%)
Liaison [c]	4.6% (3.6%–5.3%)	10.0% (8.1%–11.5%)	23.0% (19.1%–25.5%)	32.6% (27.5%–35.5%)	43.8% (37.1%–47.0%)
LumiraDX [b]	4.7% (3.7%–5.5%)	10.3% (8.3%–11.8%)	23.8% (19.7%–26.1%)	33.7% (28.4%–36.4%)	45.4% (38.4%–48.2%)
LumiraDX [c]	4.8% (3.8%–5.5%)	10.4% (8.5%–11.8%)	24.0% (20.0%–26.2%)	34.0% (28.8%–36.4%)	45.6% (39.0%–48.2%)
Omnia	4.3% (3.3%–4.9%)	9.3% (7.4%–10.7%)	21.5% (17.5%–24.0%)	30.4% (25.0%–33.4%)	40.8% (33.8%–44.3%)
SCoV-2 Ag Detect	4.2% (3.3%–4.9%)	9.1% (7.2%–10.7%)	20.9% (17.2%–23.7%)	29.7% (24.7%–33.2%)	40.0% (33.3%–44.1%)
Simoa	4.7%	10.2%	23.6%	33.4%	44.9%

	(3.8%–5.4%)	(8.4%–11.6%)	(19.8%–25.8%)	(28.3%–36.0%)	(38.5%–47.7%)
Sofia	4.6%<br>(3.6%–5.4%)	10.1%<br>(8.1%–11.6%)	23.2%<br>(19.1%–25.7%)	32.8%<br>(27.4%–35.8%)	44.1%<br>(37.0%–47.4%)
Sofia 2 Flu+SARS	4.6%<br>(3.6%–5.3%)	10.0%<br>(8.0%–11.3%)	23.1%<br>(19.1%–25.2%)	32.9%<br>(27.6%–35.4%)	44.4%<br>(37.7%–47.1%)
Status COVID-19/Flu	4.5%<br>(3.5%–5.2%)	9.7%<br>(7.8%–11.1%)	22.4%<br>(18.5%–24.9%)	31.8%<br>(26.8%–34.7%)	42.9%<br>(36.4%–46.1%)
Vitros	3.8%<br>(2.7%–4.7%)	8.3%<br>(6.1%–10.1%)	19.2%<br>(14.4%–22.7%)	27.2%<br>(20.5%–31.6%)	36.5%<br>(27.5%–41.8%)

[a] 95% credible interval
[b] Anterior nasal swab
[c] Nasopharyngeal swab

Supplementary Table 4: The probability post-travel transmission without pre-departure testing and with pre-departure testing using RT-PCR, one of the 18 rapid antigen tests, an incubation period of 4.4 days, and a basic reproduction number of 5.08. (United States, 2021–2022)

Test	Hours pre-departure					
	No test	72	48	24	12	0
RT-PCR	38.1% (37.9%–38.4%)[a]	37.5% (37.1%–37.9%)	36.7% (36.2%–37.2%)	34.5% (34.0%–35.3%)	32.6% (32.0%–33.7%)	30.0% (29.3%–31.6%)
BD Veritor	38.1% (37.9%–38.4%)	37.6% (37.2%–38.0%)	36.9% (36.5%–37.4%)	35.1% (34.5%–35.9%)	33.5% (32.8%–34.7%)	31.4% (30.4%–33.1%)
BinaxNOW	38.1% (37.9%–38.4%)	37.6% (37.2%–38.0%)	36.9% (36.5%–37.4%)	35.1% (34.5%–35.8%)	33.6% (32.9%–34.5%)	31.5% (30.6%–32.9%)
CareStart [b]	38.1% (37.9%–38.4%)	37.6% (37.2%–37.9%)	36.9% (36.4%–37.3%)	35.0% (34.3%–35.8%)	33.4% (32.6%–34.5%)	31.3% (30.2%–32.8%)
CareStart [c]	38.1% (37.9%–38.4%)	37.5% (37.2%–37.9%)	36.7% (36.3%–37.2%)	34.6% (34.1%–35.4%)	32.7% (32.2%–33.8%)	30.2% (29.5%–31.7%)
Celltrion DiaTrust	38.1% (37.9%–38.4%)	37.5% (37.1%–37.9%)	36.7% (36.3%–37.2%)	34.6% (34.1%–35.4%)	32.8% (32.2%–33.8%)	30.3% (29.5%–31.8%)
Clip COVID	38.1% (37.9%–38.4%)	37.5% (37.2%–37.9%)	36.7% (36.3%–37.2%)	34.6% (34.1%–35.4%)	32.8% (32.2%–33.9%)	30.4% (29.5%–32.0%)
Ellume	38.1% (37.9%–38.4%)	37.5% (37.1%–37.9%)	36.7% (36.3%–37.2%)	34.5% (34.0%–35.3%)	32.6% (32.1%–33.7%)	30.0% (29.3%–31.6%)
Liaison [b]	38.1% (37.9%–38.4%)	37.5% (37.2%–37.9%)	36.7% (36.3%–37.2%)	34.6% (34.1%–35.4%)	32.8% (32.2%–33.9%)	30.3% (29.5%–32.0%)
Liaison [c]	38.1% (37.9%–38.4%)	37.5% (37.2%–37.9%)	36.7% (36.3%–37.2%)	34.7% (34.1%–35.5%)	32.9% (32.2%–34.0%)	30.4% (29.6%–32.0%)
LumiraDX [b]	38.1% (37.9%–38.4%)	37.5% (37.1%–37.9%)	36.7% (36.2%–37.2%)	34.5% (34.0%–35.3%)	32.7% (32.0%–33.8%)	30.1% (29.3%–31.7%)
LumiraDX [c]	38.1% (37.9%–38.4%)	37.5% (37.1%–37.9%)	36.7% (36.2%–37.2%)	34.5% (34.0%–35.3%)	32.6% (32.0%–33.7%)	30.0% (29.3%–31.6%)
Omnia	38.1% (37.9%–38.4%)	37.6% (37.2%–37.9%)	36.8% (36.4%–37.3%)	34.9% (34.4%–35.7%)	33.3% (32.6%–34.4%)	31.1% (30.3%–32.7%)
SCoV-2 Ag Detect	38.1% (37.9%–38.4%)	37.6% (37.2%–37.9%)	36.9% (36.4%–37.4%)	35.0% (34.4%–35.8%)	33.4% (32.7%–34.5%)	31.3% (30.3%–32.8%)
Simoa	38.1% (37.9%–38.4%)	37.5% (37.1%–37.9%)	36.7% (36.3%–37.2%)	34.6% (34.0%–35.3%)	32.7% (32.1%–33.8%)	30.2% (29.4%–31.7%)

Sofia	38.1% (37.9%–38.4%)	37.5% (37.1%–37.9%)	36.7% (36.3%–37.2%)	34.6% (34.1%–35.4%)	32.8% (32.1%–34.0%)	30.4% (29.5%–32.1%)
Sofia 2 Flu+SARS	38.1% (37.9%–38.4%)	37.5% (37.2%–37.9%)	36.7% (36.3%–37.2%)	34.6% (34.1%–35.4%)	32.8% (32.2%–33.9%)	30.3% (29.6%–31.9%)
Status COVID-19/Flu	38.1% (37.9%–38.4%)	37.5% (37.2%–37.9%)	36.8% (36.4%–37.3%)	34.8% (34.2%–35.5%)	33.0% (32.4%–34.1%)	30.7% (29.8%–32.2%)
Vitros	38.1% (37.9%–38.4%)	37.6% (37.2%–38.0%)	37.0% (36.5%–37.5%)	35.3% (34.6%–36.2%)	33.9% (33.0%–35.2%)	32.1% (30.8%–33.9%)

[a] 95% credible interval
[b] Anterior nasal swab
[c] Nasopharyngeal swab

Supplementary Table 5: Reduction in the expected post-arrival transmission as a result of pre-arrival testing relative to no pre-arrival testing for RT-PCR testing, 18 rapid antigen tests and an incubation period of 5.72 days. (United States, 2021–2022)

Test	Hours pre-departure				
	72	48	24	12	0
RT-PCR	6.7%<br>(5.4%–7.7%) [a]	14.4%<br>(11.9%–16.1%)	27.4%<br>(23.0%–29.7%)	35.7%<br>(30.4%–38.2%)	45.0%<br>(38.6%–47.6%)
BD Veritor	5.6%<br>(4.3%–6.6%)	12.0%<br>(9.4%–13.9%)	23.4%<br>(18.4%–26.3%)	30.9%<br>(24.3%–34.4%)	39.3%<br>(31.0%–43.2%)
BinaxNOW	5.7%<br>(4.5%–6.7%)	12.2%<br>(9.8%–14.1%)	23.4%<br>(19.3%–26.2%)	30.6%<br>(25.4%–33.9%)	38.7%<br>(32.3%–42.4%)
CareStart [b]	5.8%<br>(4.5%–7.0%)	12.5%<br>(9.8%–14.6%)	24.0%<br>(19.5%–27.2%)	31.5%<br>(26.0%–35.1%)	39.8%<br>(33.1%–44.0%)
CareStart [c]	6.4%<br>(5.2%–7.4%)	13.9%<br>(11.5%–15.6%)	26.8%<br>(22.7%–29.0%)	35.1%<br>(29.8%–37.5%)	44.3%<br>(37.9%–46.8%)
Celltrion DiaTrust	6.4%<br>(5.2%–7.5%)	13.9%<br>(11.4%–15.6%)	26.7%<br>(22.4%–29.0%)	34.9%<br>(29.4%–37.4%)	44.0%<br>(37.4%–46.7%)
Clip COVID	6.5%<br>(5.2%–7.5%)	13.9%<br>(11.2%–15.7%)	26.5%<br>(22.0%–29.1%)	34.6%<br>(28.7%–37.5%)	43.6%<br>(36.5%–46.8%)
Ellume	6.7%<br>(5.3%–7.6%)	14.3%<br>(11.9%–16.0%)	27.3%<br>(22.9%–29.6%)	35.7%<br>(30.4%–38.0%)	44.9%<br>(38.5%–47.4%)
Liaison [b]	6.5%<br>(5.2%–7.5%)	13.9%<br>(11.3%–15.7%)	26.6%<br>(22.1%–29.1%)	34.6%<br>(28.8%–37.5%)	43.6%<br>(36.6%–46.8%)
Liaison [c]	6.4%<br>(5.1%–7.5%)	13.8%<br>(11.3%–15.7%)	26.3%<br>(22.0%–28.9%)	34.3%<br>(28.9%–37.2%)	43.2%<br>(36.6%–46.4%)
LumiraDX [b]	6.6%<br>(5.3%–7.7%)	14.2%<br>(11.6%–16.1%)	27.2%<br>(22.6%–29.7%)	35.5%<br>(29.8%–38.1%)	44.7%<br>(37.8%–47.5%)
LumiraDX [c]	6.7%<br>(5.4%–7.7%)	14.4%<br>(11.8%–16.1%)	27.4%<br>(22.9%–29.7%)	35.7%<br>(30.4%–38.2%)	45.0%<br>(38.4%–47.6%)
Omnia	6.0%<br>(4.7%–7.0%)	12.8%<br>(10.3%–14.7%)	24.5%<br>(20.1%–27.2%)	32.0%<br>(26.3%–35.1%)	40.3%<br>(33.3%–43.7%)
SCoV-2 Ag Detect	5.8%<br>(4.6%–7.0%)	12.5%<br>(10.0%–14.5%)	23.9%<br>(19.7%–26.9%)	31.3%<br>(25.9%–34.8%)	39.4%<br>(32.8%–43.5%)
Simoa	6.6%	14.1%	26.9%	35.1%	44.2%

	(5.3%–7.6%)	(11.7%–15.8%)	(22.7%–29.3%)	(29.8%–37.8%)	(37.9%–47.1%)
Sofia	6.5% (5.1%–7.6%)	13.9% (11.2%–15.8%)	26.5% (21.9%–29.2%)	34.5% (28.8%–37.5%)	43.5% (36.4%–46.8%)
Sofia 2 Flu+SARS	6.4% (5.0%–7.3%)	13.7% (11.2%–15.4%)	26.4% (22.1%–28.8%)	34.6% (29.1%–37.2%)	43.7% (37.2%–46.5%)
Status COVID-19/Flu	6.2% (4.9%–7.3%)	13.4% (10.8%–15.2%)	25.6% (21.4%–28.2%)	33.5% (28.3%–36.5%)	42.3% (35.9%–45.5%)
Vitros	5.3% (3.9%–6.6%)	11.5% (8.5%–13.8%)	21.9% (16.5%–25.8%)	28.6% (21.6%–33.2%)	36.0% (27.1%–41.3%)

[a] 95% credible interval
[b] Anterior nasal swab
[c] Nasopharyngeal swab

Supplementary Table 6: The probability post-travel transmission without pre-departure testing and with pre-departure testing using RT-PCR, one of the 18 rapid antigen tests, an incubation period of 5.72 days, and a basic reproduction number of 2.79. (United States, 2021–2022)

Test	Hours pre-departure					
	No test	72	48	24	12	0
RT-PCR	31.8% (31.5%–32.1%)[a]	30.9% (30.5%–31.3%)	29.7% (29.3%–30.3%)	27.6% (27.0%–28.4%)	26.0% (25.4%–27.1%)	24.0% (23.3%–25.4%)
BD Veritor	31.8% (31.5%–32.1%)	31.0% (30.7%–31.5%)	30.1% (29.6%–30.6%)	28.3% (27.6%–29.2%)	26.9% (26.2%–28.1%)	25.2% (24.3%–26.9%)
BinaxNOW	31.8% (31.5%–32.1%)	31.0% (30.6%–31.4%)	30.1% (29.6%–30.6%)	28.3% (27.7%–29.1%)	27.0% (26.3%–28.0%)	25.4% (24.5%–26.7%)
CareStart [b]	31.8% (31.5%–32.1%)	31.0% (30.6%–31.4%)	30.0% (29.5%–30.6%)	28.2% (27.5%–29.0%)	26.8% (26.0%–27.9%)	25.1% (24.2%–26.6%)
CareStart [c]	31.8% (31.5%–32.1%)	30.9% (30.5%–31.4%)	29.8% (29.4%–30.4%)	27.7% (27.1%–28.5%)	26.1% (25.5%–27.2%)	24.1% (23.5%–25.5%)
Celltrion DiaTrust	31.8% (31.5%–32.1%)	30.9% (30.5%–31.3%)	29.8% (29.4%–30.4%)	27.7% (27.2%–28.5%)	26.1% (25.5%–27.2%)	24.2% (23.5%–25.7%)
Clip COVID	31.8% (31.5%–32.1%)	30.9% (30.5%–31.3%)	29.8% (29.4%–30.4%)	27.7% (27.1%–28.6%)	26.2% (25.5%–27.4%)	24.3% (23.5%–25.9%)
Ellume	31.8% (31.5%–32.1%)	30.9% (30.5%–31.3%)	29.8% (29.3%–30.3%)	27.6% (27.1%–28.4%)	26.0% (25.4%–27.1%)	24.0% (23.3%–25.4%)
Liaison [b]	31.8% (31.5%–32.1%)	30.9% (30.5%–31.3%)	29.8% (29.4%–30.4%)	27.7% (27.1%–28.6%)	26.2% (25.5%–27.4%)	24.3% (23.5%–25.8%)
Liaison [c]	31.8% (31.5%–32.1%)	30.9% (30.5%–31.4%)	29.8% (29.4%–30.4%)	27.8% (27.2%–28.6%)	26.3% (25.6%–27.4%)	24.4% (23.6%–25.9%)
LumiraDX [b]	31.8% (31.5%–32.1%)	30.9% (30.5%–31.3%)	29.8% (29.3%–30.3%)	27.6% (27.0%–28.5%)	26.0% (25.4%–27.2%)	24.0% (23.3%–25.6%)
LumiraDX [c]	31.8% (31.5%–32.1%)	30.9% (30.5%–31.3%)	29.7% (29.3%–30.3%)	27.6% (27.0%–28.4%)	26.0% (25.4%–27.1%)	24.0% (23.3%–25.4%)
Omnia	31.8% (31.5%–32.1%)	31.0% (30.6%–31.4%)	30.0% (29.5%–30.5%)	28.1% (27.5%–29.0%)	26.7% (26.0%–27.8%)	25.0% (24.2%–26.5%)
SCoV-2 Ag Detect	31.8% (31.5%–32.1%)	31.0% (30.6%–31.4%)	30.0% (29.5%–30.6%)	28.2% (27.5%–29.0%)	26.8% (26.1%–27.9%)	25.2% (24.3%–26.6%)
Simoa	31.8% (31.5%–32.1%)	30.9% (30.5%–31.3%)	29.8% (29.3%–30.3%)	27.7% (27.1%–28.5%)	26.1% (25.5%–27.2%)	24.2% (23.4%–25.6%)
Sofia	31.8%	30.9%	29.8%	27.7%	26.2%	24.3%

	(31.5%–32.1%)	(30.5%–31.4%)	(29.3%–30.4%)	(27.1%–28.6%)	(25.5%–27.4%)	(23.5%–25.9%)
Sofia 2 Flu+SARS	31.8% (31.5%–32.1%)	30.9% (30.5%–31.4%)	29.8% (29.4%–30.4%)	27.7% (27.2%–28.6%)	26.2% (25.6%–27.3%)	24.3% (23.6%–25.8%)
Status COVID-19/Flu	31.8% (31.5%–32.1%)	31.0% (30.6%–31.4%)	29.9% (29.4%–30.5%)	27.9% (27.3%–28.7%)	26.4% (25.8%–27.5%)	24.6% (23.8%–26.0%)
Vitros	31.8% (31.5%–32.1%)	31.1% (30.7%–31.5%)	30.2% (29.7%–30.8%)	28.5% (27.7%–29.5%)	27.3% (26.4%–28.7%)	25.9% (24.7%–27.6%)

[a] 95% credible interval

[b] Anterior nasal swab

[c] Nasopharyngeal swab

Supplementary Table 7: The estimated coefficients from the linear logit models of the percent positive agreement. (United States, 2021–2022)

Test Name	β_0	β_1
BD Veritor	2.55<br>(1.22–4.82) [a]	-3.79×10^{-1}<br>$(-8.51 \times 10^{-1}\text{–}-7.78 \times 10^{-2})$
BinaxNOW	1.95<br>(1.30–2.89)	-1.00×10^{-1}<br>$(-2.14 \times 10^{-1}\text{–}-1.65 \times 10^{-2})$
CareStart [b]	2.20<br>(1.52–4.05)	-1.23×10^{-1}<br>$(-5.93 \times 10^{-1}\text{–}-1.17 \times 10^{-2})$
CareStart [c]	6.77<br>(3.34–22.57)	-9.98×10^{-1}<br>$(-4.22\text{–}-1.45 \times 10^{-1})$
Celltrion DiaTrust	4.81<br>(2.57–12.27)	-5.18×10^{-1}<br>$(-1.71\text{–}-5.31 \times 10^{-2})$
Clip COVID	3.43<br>(1.94–6.94)	-1.28×10^{-8}<br>$(-6.23 \times 10^{-8}\text{–}-1.70 \times 10^{-9})$
Ellume	100.00<br>(73.75–485.25)	-15.3<br>$(-75.3\text{–}-11.3)$
Liaison [b]	3.47<br>(1.98–6.97)	-1.15×10^{-9}<br>$(-5.58 \times 10^{-9}\text{–}-1.29 \times 10^{-10})$
Liaison [c]	3.20<br>(2.15–5.17)	-1.73×10^{-9}<br>$(-8.51 \times 10^{-9}\text{–}-1.99 \times 10^{-10})$
LumiraDX [b]	5.64<br>(3.74–11.41)	-3.27×10^{-1}<br>$(-8.79 \times 10^{-1}\text{–}-3.60 \times 10^{-2})$
LumiraDX [c]	12.09<br>(6.01–48.89)	-1.05<br>$(-4.60\text{–}-3.17 \times 10^{-1})$
Omnia	2.14<br>(1.43–3.10)	-1.81×10^{-9}<br>$(-8.79 \times 10^{-9}\text{–}-3.12 \times 10^{-10})$
SCoV-2 Ag Detect	2.03<br>(1.41–3.36)	-5.37×10^{-2}<br>$(-2.62 \times 10^{-1}\text{–}-6.17 \times 10^{-3})$
Simoa	4.17<br>(3.09–7.24)	-7.78×10^{-2}<br>$(-3.59 \times 10^{-1}\text{–}-6.03 \times 10^{-3})$
Sofia	3.37	-8.89×10^{-10}

	(2.05–6.97)	(-4.38×10^{-9}–-1.07×10^{-10})
Sofia 2 Flu+SARS	4.77 (2.81–12.31)	-6.00×10^{-1} (-2.36–-4.95×10^{-2})
Status COVID-19/Flu	2.93 (2.13–4.96)	-1.35×10^{-1} (-6.48×10^{-1}–-1.01×10^{-2})
Vitros	1.39 (0.58–2.47)	-8.00×10^{-10} (-3.90×10^{-9}–-9.60×10^{-11})

[a] 95% credible interval
[b] Anterior nasal swab
[c] Nasopharyngeal swab

Supplementary References

1. Hellewell J, Russell TW, SAFER Investigators and Field Study Team, Crick COVID-19 Consortium, CMMID COVID-19 working group, Beale R, et al. Estimating the effectiveness of routine asymptomatic PCR testing at different frequencies for the detection of SARS-CoV-2 infections. Vol. 19, BMC medicine. 2021. p. 106. Available from: http://dx.doi.org/10.1186/s12916-021-01982-x
